# Increased CO_2_ Affinity and Adsorption Selectivity in MOF-801 Fluorinated Analogues

**DOI:** 10.1021/acsami.2c07640

**Published:** 2022-08-30

**Authors:** Diletta Morelli Venturi, Maria Sole Notari, Roberto Bondi, Edoardo Mosconi, Waldemar Kaiser, Giorgio Mercuri, Giuliano Giambastiani, Andrea Rossin, Marco Taddei, Ferdinando Costantino

**Affiliations:** † Department of Chemistry, Biology and Biotechnology, Università degli Studi di Perugia, via Elce di Sotto, 8, 06123 Perugia, Italy; ‡ Computational Laboratory for Hybrid/Organic Photovoltaics (CLHYO), Istituto CNR di Scienze e Tecnologie Chimiche “Giulio Natta” (CNR-SCITEC), Via Elce di Sotto 8, 06123 Perugia, Italy; § Istituto di Chimica dei Composti Organometallici (CNR-ICCOM), Via Madonna del Piano 10, 50019 Sesto Fiorentino, Firenze, Italy; ∥ Scuola del Farmaco e dei Prodotti della Salute, Università di Camerino, Via S. Agostino 1, 62032 Camerino, Italy; ⊥ Department of Chemistry and Industrial Chemistry, University of Pisa, Via Giuseppe Moruzzi 13, 56124 Pisa, Italy

**Keywords:** metal−organic frameworks (MOFs), carbon dioxide capture, zirconium, fluorinated linkers, DFT calculations

## Abstract

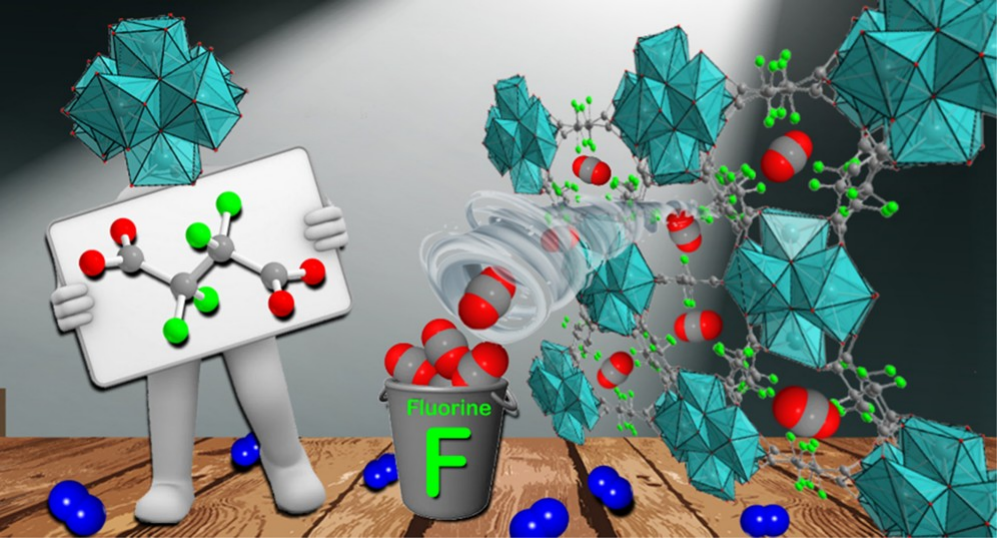

The novel Zr^IV^-based perfluorinated metal–organic framework (PF-MOF) [Zr_6_O_4_(OH)_4_(**TFS**)_6_] (**ZrTFS**) was prepared under solvent-free conditions using the commercially available tetrafluorosuccinic acid (**H**_**2**_**TFS**) as a bridging ditopic linker. Since **H**_**2**_**TFS** can be seen as the fully aliphatic and perfluorinated C_4_ analogue of fumaric acid, **ZrTFS** was found to be isoreticular to zirconium fumarate (**MOF-801**). The structure of **ZrTFS** was solved and refined from X-ray powder diffraction data. Despite this analogy, the gas adsorption capacity of **ZrTFS** is much lower than that of **MOF-801**; in the former, the presence of bulky fluorine atoms causes a considerable window size reduction. To have PF-MOFs with more accessible porosity, postsynthetic exchange (PSE) reactions on (defective) **MOF-801** suspended in **H**_**2**_**TFS** aqueous solutions were carried out. Despite the different **H**_**2**_**TFS** concentrations used in the PSE process, the exchanges yielded two mixed-linker materials of similar minimal formulae [Zr_6_O_4_(μ_3_-OH)_4_(μ_1_-OH)_2.08_(H_2_O)_2.08_(**FUM**)_4.04_(**HTFS**)_1.84_] (**PF-MOF1**) and [Zr_6_O_4_(μ_3_-OH)_4_(μ_1_-OH)_1.83_(H_2_O)_1.83_(**FUM**)_4.04_(**HTFS**)_2.09_] (**PF-MOF2**) (**FUM**^**2–**^ = fumarate), where the perfluorinated linker was found to fully replace the capping acetate in the defective sites of pristine **MOF-801**. CO_2_ and N_2_ adsorption isotherms collected on all samples reveal that both CO_2_ thermodynamic affinity (isosteric heat of adsorption at zero coverage, *Q*_st_) and CO_2_/N_2_ adsorption selectivity increase with the amount of incorporated **TFS**^**2–**^, reaching the maximum values of 30 kJ mol^–1^ and 41 (IAST), respectively, in **PF-MOF2**. This confirms the beneficial effect coming from the introduction of fluorinated linkers in MOFs on their CO_2_ adsorption ability. Finally, solid-state density functional theory calculations were carried out to cast light on the structural features and on the thermodynamics of CO_2_ adsorption in **MOF-801** and **ZrTFS**. Due to the difficulties in modeling a defective MOF, an intermediate structure containing both linkers in the framework was also designed. In this structure, the preferential CO_2_ adsorption site is the tetrahedral pore in the “UiO-66-like” structure. The extra energy stabilization stems from a hydrogen bond interaction between CO_2_ and a hydroxyl group on the inorganic cluster.

## Introduction

The urgent need to reduce CO_2_ emissions is pushing scientists and engineers toward the development of technologies that should prevent further buildup of this greenhouse gas in the atmosphere. Among the technologies under scrutiny, carbon capture and storage (CCS)^1^ from large point sources and direct air capture (DAC)^2^ are seen as viable options for rapid large-scale deployment. The state-of-the-art sorbents for CCS and DAC are typically amine-based: aqueous methanolamine solutions for CCS^3^ and solid-supported amine adsorbents for DAC.^4^ In both cases, the working principle exploits the formation of covalent bonds between the amine groups and CO_2_ to afford carbamates (chemical adsorption or chemisorption). The formation of strong covalent bonds is associated with a high heat of absorption or adsorption, which is convenient to achieve high capture loading at low partial pressures but poses challenges in terms of regeneration of the sorbent and achievable working capacity.^5,6^ While DAC demands a sorbent with a high enthalpy of ab/adsorption, due to the large entropic loss associated with CO_2_ capture from a feed that contains only ∼415 ppm of CO_2_, CCS deals with CO_2_ concentrations in the range between 4 and 30% and could greatly benefit from a sorbent with a mild heat of adsorption, i.e., a physisorbent.^7^ To this end, adsorbents such as porous carbons,^8^ zeolites,^9^ and metal–organic frameworks (MOFs)^5−7^ are intensely investigated.

The outstanding versatility of MOFs in terms of pore size, shape, and chemistry has led to the development of a large number of sorbents that display remarkable CO_2_ capture performance,^10−14^ recently culminated with the deployment of CALF-20 for capture in the cement industry.^15^ Fine tuning of pore size and shape is crucial to maximize the framework–adsorbate contact.^16^ This has recently led ultramicroporous MOFs—i.e., with pores smaller than 8 Å—to gain a privileged spot as promising sorbents for CO_2_ capture.^17−19^ Pore chemistry plays a key role at the low partial pressures relevant for CCS, where specific interactions with the framework dominate the adsorption process. Typical CO_2_ adsorption sites in MOFs include coordinatively unsaturated metal ions and functional groups with a basic character on the organic linker.^7,11,12^ A promising approach that is gaining momentum is to introduce fluorine atoms in the backbone of ultramicroporous MOFs to prepare new materials labeled as perfluorinated MOFs (PF-MOFs).^20,21^ Besides increasing the thermodynamic affinity for CO_2_ (as evidenced by a high isosteric heat of adsorption, *Q*_st_), the presence of (per)fluorinated groups can also render the framework hydrophobic, thus preserving the CO_2_ capture performance in humid conditions, which is crucial for real-life applications. The family of hybrid ultramicroporous materials containing fluorinated anions such as SiF_6_^2–^, NbOF_5_^2–^, and TiF_6_^2–^ represents an excellent example of how the presence of fluorine atoms exposed in narrow pores provides superior affinity for CO_2_ while preserving a physisorption-based mechanism and minimizing competitive water adsorption.^20,22−24^ We have recently reported on the water-based synthesis of two perfluorinated Ce^IV^ analogues of the well-known UiO-66 and MIL-140A framework types.^21,25^ The ultramicroporous F4-MIL-140A(Ce) displays an S-shaped isotherm and outstanding CO_2_/N_2_ selectivity, and it was also found to display inverse CO_2_/C_2_H_2_ selectivity.^26^

**Scheme 1:**
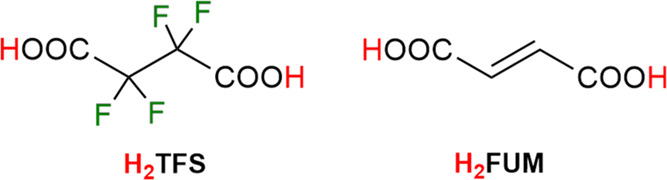
Linkers Used in This Study for the Construction of PF-MOFs

As a result of our continued effort in developing highly stable (per)fluorinated MOFs based on tetravalent metals for CCS applications,^27^ we report herein on the preparation and solid-state characterization of a perfluorinated analogue of the well-known **MOF-801**, namely, Zr^IV^ tetrafluorosuccinate (**ZrTFS**), and of the two mixed-linker perfluorinated MOFs (PF-MOFs) **PF-MOF1** and **PF-MOF2** through postsynthetic tetrafluorosuccinate/fumarate (Scheme 1) linker exchange starting from pure zirconium fumarate **MOF-801**. The relationship between CO_2_ adsorption capacity/selectivity and the extent of fluorination in solid adsorbents has been systematically studied.

## Experimental Section

### Materials and Methods

All chemicals are commercially available and used as received from the specified vendors without further purification. Zirconium chloride (ZrCl_4_) was purchased from Alfa Aesar. Tetrafluorosuccinic acid (**H**_**2**_**TFS**) was purchased from Fluorochem. Fumaric acid (**H**_**2**_**FUM**) was purchased from Merck. Acetic acid (AA) was purchased from Carlo Erba. Powder X-ray diffraction (PXRD) patterns were collected in reflection geometry in the 4–40° 2θ range, with a 40 s step^–1^ counting time and with a step size of 0.016° on a PANalytical X’PERT PRO diffractometer, a PW3050 goniometer, equipped with an X’Celerator detector and using a Cu Kα radiation source. The long fine focus (LFF) ceramic tube operated at 40 kV and 40 mA. Quantitative ^1^H NMR and ^19^F NMR analyses were performed at an ambient temperature on either a Bruker Ascend 400 MHz spectrometer (**MOF-801**) or a Jeol JNM-ECZ500S instrument equipped with a RoyalProbe Broadband probe (**PF-MOF1** and **PF-MOF2**): ^1^H: acquisition time 4.36 s, relaxation delay τ = 25 s, 4 scans; ^19^F: acquisition time 0.27 s, relaxation delay τ = 4 s, 16 scans. The solids (ca. 20 mg) were held in an oven at 120 °C for 2 h before being digested for 24 h in 1.5 mL of 1 M NaOH in D_2_O (**MOF-801**) or in 1.0 mL of 1 M NaOH in D_2_O (**PF-MOF1** and **PF-MOF2**). The NMR tubes were then loaded with the solution, which was filtered through cotton wool to avoid the presence of solid particles in suspension. 3-Fluorobenzoic acid (0.029 M) was used as the ^1^H internal standard for the fumarate and acetate quantification in **MOF-801**. 2,6-Difluorobenzoic acid (0.1 M) was used as both ^1^H and ^19^F internal standards for the fumarate and tetrafluorosuccinate quantification in the mixed-linker MOFs (see the Supporting Information for details on the calculations). Thermogravimetric analysis (TGA) was performed using a Netzsch STA490C thermoanalyzer under a 20 mL min^–1^ air flux with a heating rate of 10 °C min^–1^. Transmission Fourier transform infrared spectroscopy (FT-IR) spectra (KBr pellets) were recorded on a Perkin-Elmer Spectrum BX Series FT-IR spectrometer, in the 4000–400 cm^–1^ range, with a 2 cm^–1^ resolution. Inductively coupled plasma optical emission spectroscopy (ICP-OES) analysis was carried out using a Varian 700-ES series. A calibration curve was obtained with four standard zirconium solutions (0.5, 1, 2.5, and 5 mg L^–1^, respectively). The analyses were performed on the supernatant after dissolving the solids in an HNO_3_ (2%) aqueous solution. Scanning electron microscopy (SEM) images were acquired with an FEI Quanta 450 ESEM FEG, working at a 15.00 kV acceleration voltage. The samples were sputtered with graphite prior to the analysis.

### Synthesis of [Zr_6_O_4_(OH)_4_(**TFS**)_6_] (Zirconium Tetrafluorosuccinate, **ZrTFS**)

ZrCl_4_ (1 mmol, 233 mg), **H**_**2**_**TFS** (4 mmol, 773 mg), and 1 mL of deionized water were put in a ball-mill vessel. After 15 min of milling, the mixture was put in a 5 mL hydrothermal bomb and was heated to 120 °C for 72 h. The obtained white solid was recovered by centrifugation and washed with ethanol (2 × 5 mL), water (1 × 5 mL), and acetone (2 × 5 mL) and finally dried in an oven at 80 °C. Yield: 267 mg (88%, based on zirconium). IR (KBr, cm^–1^; Figure S1): 1663(s) [ν(COO^–^)], 1145/1128 (m) [ν(C–F)_sym/asym_].

### Synthesis of [Zr_6_O_4_(μ_3_-OH)_4_(μ_1_-OH)_3.12_(H_2_O)_3.12_(**FUM**)_4.04_(AA)_0.80_] (Zirconium Fumarate, **MOF-801**)

ZrCl_4_ (1 mmol, 233 mg), **H**_**2**_**FUM** (3 mmol, 348 mg), and acetic acid (100 mmol, 5.7 mL) were placed together in a hydrothermal bomb with 20 mL of deionized water, and the solution was sonicated until complete reagent dissolution. Then, the reactor was held in a thermostatic oven at 120 °C for 24 h. The obtained white solid was recovered by centrifugation, washed with water (2 × 10 mL) and acetone (1 × 10 mL), and finally dried in an oven at 80 °C. Yield: 200 mg (87%, based on zirconium). The phase identity and purity were checked through PXRD. The amount of AA present in the material was estimated through ^1^H NMR (see the Supporting Information). IR (KBr, cm^–1^; Figure S1): 1576(s) [ν(C=C)_FUM_]. The [ν(COO^–^)_FUM_] and [ν(COO^–^)_AA_] bands are not visible, falling underneath that of [ν(C=C)_FUM_].

### Postsynthetic Exchange on **MOF-801**: Preparation of [Zr_6_O_4_(μ_3_-OH)_4_(μ_1_-OH)_2.08_(H_2_O)_2.08_(**FUM**)_4.04_(**HTFS**)_1.84_] (**PF-MOF1**) and [Zr_6_O_4_(μ_3_-OH)_4_(μ_1_-OH)_1.83_(H_2_O)_1.83_(**FUM**)_4.04_(**HTFS**)_2.09_] (**PF-MOF2**)

**MOF-801** (200 mg, 0.14 mmol) and **H**_**2**_**TFS** (0.28 mmol, 68 mg for the synthesis of **PF-MOF1** and 0.84 mmol, 160 mg for the synthesis of **PF-MOF2**, respectively) were placed together in a reaction flask with 2.5 mL of deionized water. The mixture was left under stirring at 60 °C for 4 h. The obtained solids were recovered by centrifugation, washed with ethanol (2 × 5 mL), water (1 × 5 mL), and acetone (1 × 5 mL), and finally dried in an oven at 80 °C. Yield: 224 mg and 237 mg (99.99 and 99.93%, based on zirconium present in the pristine MOF-801 and using the results from ICP measurements for quantifying the metal leaching after the exchange; Table S1) for **PF-MOF1** and **PF-MOF2**, respectively. The proposed minimal formulae reported above come from quantitative ^1^H and ^19^F NMR spectroscopies after digestion and TGA carried out to quantify the number of defects and the amount of exchanged **TFS**^**2–**^ ligands. **PF-MOF1**. IR (KBr, cm^–1^; Figure S1): 1645(vs) [ν(COO^–^)_TFS_], 1576 (vs) [ν(C=C)_FUM_], 1135/1118 (s) [ν(C–F)_sym/asym_]. **PF-MOF2**. IR (KBr, cm^–1^; Figure S1): 1653(vs) [ν(COO^–^)_TFS_], 1576(vs) [ν(C=C)_FUM_], 1136/1118 (s) [ν(C–F)_sym/asym_]. As found in **MOF-801**, the [ν(COO^–^)_FUM_] bands are not visible, falling underneath that of [ν(C=C)_FUM_].

### N_2_ and CO_2_ Adsorptions

All of the samples were activated at 120 °C under a high vacuum (10^–6^ Torr) for 12 h before any measurement. The Brunauer–Emmett–Teller (BET) specific surface area (SSA) and porosity were estimated by volumetric adsorption with an ASAP 2020 Micromeritics instrument, using N_2_ as the adsorbate at −196 °C and an equilibration time of 30 s (an optimal value for surface area measurements when using N_2_ as a probe with ultramicroporous materials).^28^ A typical measurement used 40 mg of sample. For the BET specific surface area calculation, the 0.01–0.1 *p*/*p*_0_ pressure range was used to fit the data. Within this range, all of the Rouquerol consistency criteria^29,30^ are satisfied. The material (micro)porosity was determined from the CO_2_ adsorption isotherms collected at *T* = 0 °C, using a 2D nonlocal density functional theory (2D-NLDFT) method successfully employed for carbonaceous materials with heterogeneous surfaces.^31^ The same kind of pore size distribution (PSD) can also be obtained from N_2_ isotherms at *T* = −196 °C.^32^ CO_2_ adsorption isotherms were measured at 0 and 25 °C up to the maximum pressure of 1.2 bar. The isosteric heat of adsorption (*Q*_st_) was calculated working on the CO_2_ isotherms measured at 0 and 25 °C, by applying a variant of the Clausius–Clapeyron equation (eq 1):^33,34^
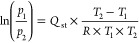
1where *p_n_* (*n* = 1 or 2) denotes the pressure value for the *n*th isotherm, *T_n_* (*n* = 1 or 2) denotes the temperature value for the *n*th isotherm, and *R* is the gas constant expressed in the appropriate units (8.314 J K^–1^ mol^–1^). In order to validate the calculated *Q*_st_ values on two temperatures, an additional isotherm was also collected at T = −20 °C for **PF-MOF1** and **ZrTFS** and the calculation was repeated on three temperature points using the differential form of the Clausius-Clapeyron equation (see the Supporting Information). The isotherms were also fitted with the Virial equation following the guidelines of a recent paper^35^
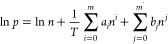
2where *p* is the pressure at which loading *n* is reached, *a*_*i*_ (*m* = 7) is −*Q*_*i*_/*R*, and *b*_*j*_ (*m*′ = 2) is a constant. *Q*_st_ was then derived using the following equation
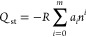
3

To evaluate the CO_2_/N_2_ selectivity at 25 °C, the N_2_ adsorption isotherms were acquired at 25 °C and up to 1.2 bar. The CO_2_/N_2_ Henry selectivity was calculated as the ratio of the initial slopes of the two adsorption isotherms in the 0.01 ≤ *p* ≤ 0.1 bar range. The CO_2_/N_2_ ideal adsorbed solution theory (IAST) selectivity of binary mixtures at a total pressure of 1 bar and at *T* = 25 °C was determined as the ratio of the adsorbed molar fractions of the two gases divided by the ratio of the gas-phase initial molar fractions^36^
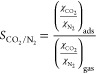
4

The (χ)_ads_ values were derived from the application of the free software pyIAST^37^ (https://github.com/CorySimon/pyIAST) to the experimental single-component isotherms collected at the chosen temperature. The initial composition (%) for the calculation was [15:85] for the [CO_2_/N_2_] pair. This relative ratio was selected to mimic the general feed composition of a coal-fired power station.^38^ A Henry or a BET model was employed for the isotherm fitting. For a detailed explanation of these models and the related parameters, see the pyIAST webpage and documentation.

### Computational Details

All calculations were carried out with the CP2K code.^39^ Atom-centered Gaussian-type basis functions are used to describe the orbitals. The MOLOPT^40^ basis set for Zr, O, C, F, and H was employed, with a cut-off of 500 Ha for the plane waves along with the PBE functional.^41^ Core–valence interactions are described by Goedecker–Teter–Hutter pseudopotentials.^42^ In this simulation, the experimental structures derived from PXRD data were taken as the initial geometry guess and a joint atomic position and cell parameter optimization was carried out, keeping the three axes orthogonal and symmetric. The Brillouin zone was sampled at the Γ point. Mulliken atomic charges on a reference model (SiF_6_^2–^) and on the **H**_**2**_**TFS** organic linker at comparison were calculated by performing a Gaussian 09^43^ geometry optimization using the B3LYP exchange–correlation functional^44^ and a 6-31++g** basis set on all atoms.

## Results and Discussion

### Synthesis and Structural Characterization of ZrTFS

**ZrTFS** was prepared with a “Shake ‘n Bake” procedure that we have previously employed for the synthesis of several UiO-66 MOF analogues.^45^ In this procedure, a dense slurry of reagents in a small amount of a liquid is obtained instead of a clear solution upon a preliminary treatment using a ball mill. This probably induces a partial MOF crystallization before the heating stage, which provides a crystalline material at the end of the synthesis. If the milling stage is bypassed, a much less crystalline product is obtained (Figure S2) and the reaction yield decreases from 88 to 69%. No solid product was obtained when larger volumes of water were used, suggesting that a high concentration of reagents is necessary to induce the crystallization of **ZrTFS**. This is likely due to the high solubility of **H**_**2**_**TFS** in water, a result of its highly acidic character (p*K*_a_ values of 1.64 and 3.68, determined using Chemicalize, https://chemicalize.com/, developed by ChemAxon). Notably, to the best of our knowledge, **ZrTFS** is only the second material with an extended three-dimensional framework built with tetrafluorosuccinic acid reported in the Cambridge Structural Database, the other one being a lithium derivative.^46^

**Figure 1:**
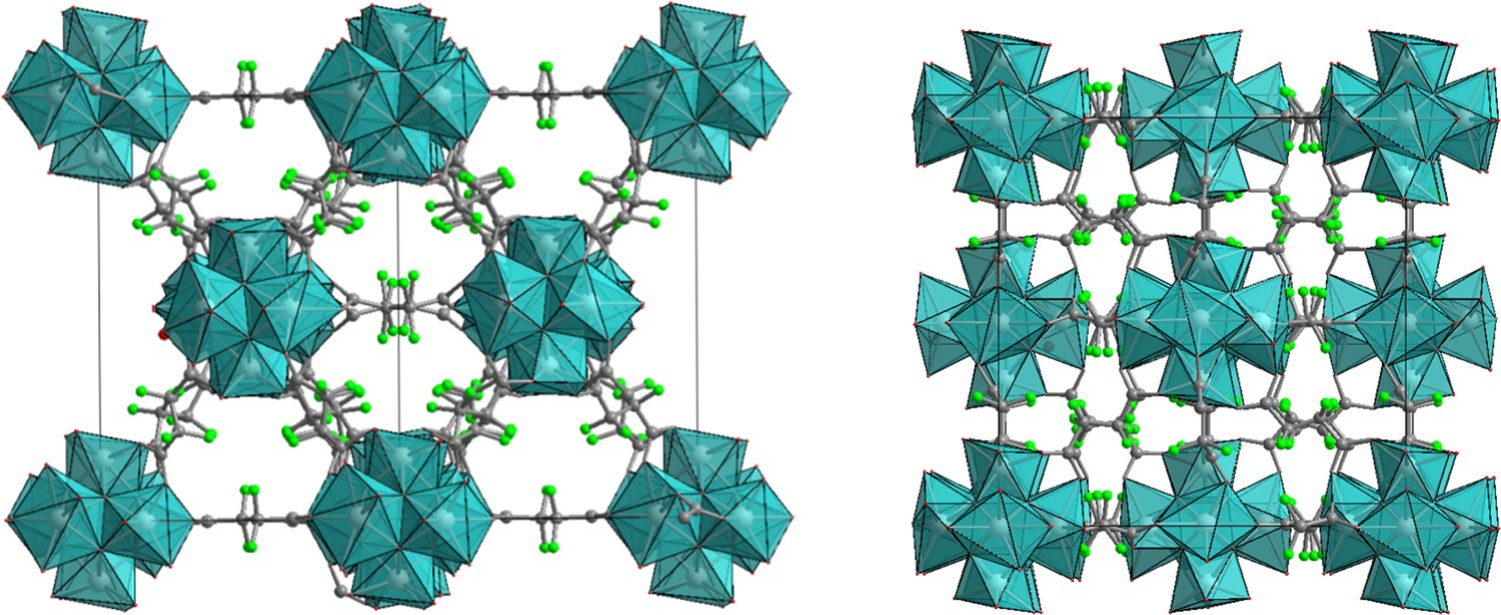
Polyhedral representation of **ZrTFS** viewed along the (110) and (100) directions. Carbon atoms are depicted in gray, fluorine atoms are depicted in green, and zirconium atoms are depicted in light blue.

The structure of **ZrTFS** was solved *ab initio* from PXRD data using the parallel tempering algorithm implemented in the FOX program^47^ and refined with the Rietveld method using TOPAS.^48^ Details of structure solution and refinement are reported in the Supporting Information (Figure S3). A polyhedral representation of the structure as obtained from the Rietveld refinement is reported in Figure 1. The structure was solved in the same cubic space group as **MOF-801** (*Pn*3̅), and it is fully consistent with the optimized DFT structure (see below). The lattice parameter is 18.0690 Å, slightly larger than that reported for **MOF-801** (17.8469 Å).^49^ The framework topology is **fcu** with 12-connected [Zr_6_O_4_(OH)_4_]^12+^ hexanuclear clusters bridged by the **TFS**^**2–**^ linkers. The geometry of one crystallographically independent linker recalls that of fumaric acid in **MOF-801** with the O–C–C–O carbon–oxygen chain torsion angle close to 180° (i.e., O11–C11–C12–C12#1 176.9° #1 = −*x*, *y* + 1/2, *z* + 1/2), whereas the second **TFS**^**2–**^ displays a bent geometry between the carboxylic plane and the C–F chain (O21–C21–C22–C22#1 146.5° #1 = −*x*, *y* + 1/2, *z* + 1/2). Fluorine atoms linked to internal carbon atoms (C–F distance 1.37 Å) are pointing toward the cavities, thus reducing the window size with respect to those of **MOF-801** from 4.8 to 3.5 Å (average distances measured from atomic centers). These small window sizes strongly limit the gas diffusion into the cavities, as observed in its gas adsorption isotherms (*vide infra*). SEM analysis (Figure S4) reveals a peculiar truncated octahedral morphology, with the crystallite size in the range of 500 nm.

### Synthesis of Mixed-Linker MOFs via Postsynthetic Exchange

**Figure 2:**
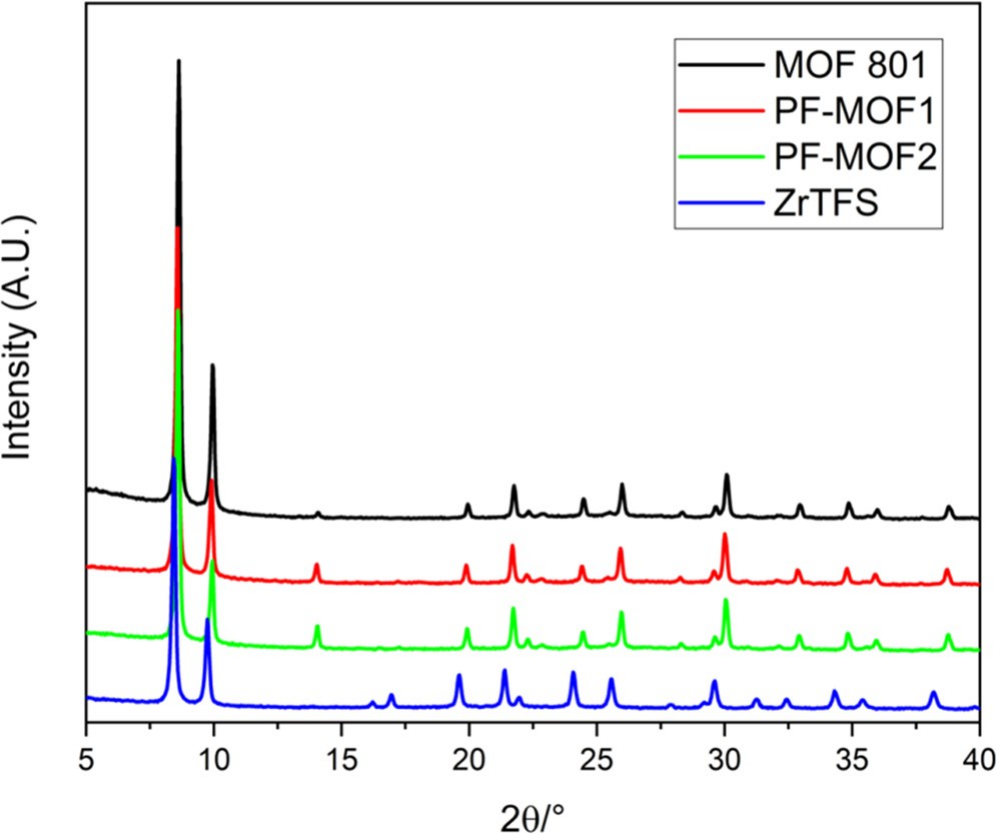
PXRD patterns of **MOF-801** (black), **PF-MOF1** (red), **PF-MOF2** (green), and **ZrTFS** (blue) in comparison.

**Figure 3:**
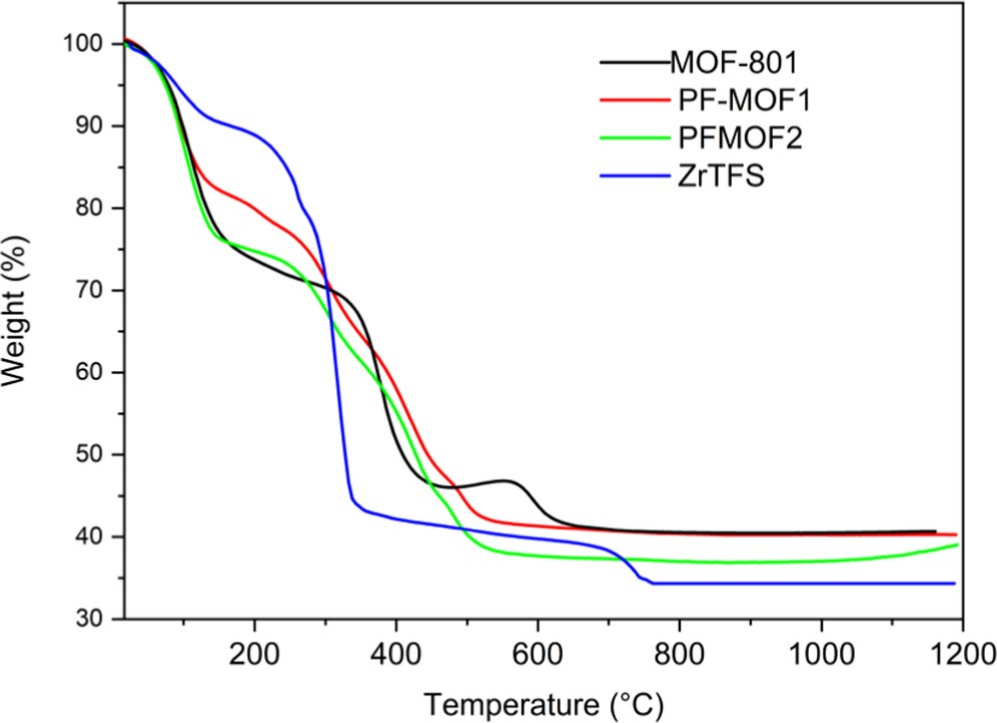
TGA profiles of **MOF-801** (black), **PF-MOF1** (red), **PF-MOF2** (green), and **ZrTFS** (blue) in comparison.

The lack of accessible porosity in the framework of **ZrTFS** limits its ability to adsorb CO_2_. This prompted us to prepare alternative materials where the (flexible) fully aliphatic **TFS**^**2–**^ linker and its (more rigid) unsaturated analogue **FUM**^**2–**^ could coexist in the same framework. Mixed-linker tetrafluorosuccinate/fumarate MOFs should feature both satisfactory accessible surface area and high fluorine content. To achieve this result, we carried out postsynthetic exchange (PSE) reactions^50−52^ starting from pure **MOF-801**.^53^**MOF-801** was synthesized from fumaric acid and Zr^IV^ chloride according to the literature procedure of Zahn et al., using acetic acid as the crystallinity modulator.^54^ Its PXRD pattern is reported in Figure 2, confirming the phase purity and the high crystallinity degree. The use of AA as a modulator leads to the obtainment of a defective material where the bridging fumarate linkers are partially replaced by terminal acetates and water/hydroxide couples. The ^1^H NMR signal integration of the digested sample using 3-fluorobenzoic acid as the internal standard (Figure S5) allowed us to quantify the ratio of fumarate (**FUM**) and acetate (AA) in the structure (calculation details are provided in the Supporting Information). The general formula found is [Zr_6_O_4_(μ_3_-OH)_4_(μ_1_-OH)_3.12_(H_2_O)_3.12_(**FUM**)_4.04_(AA)_0.80_]. From **MOF-801**, fumarate/tetrafluorosuccinate partial exchange performed in water with a (1:2) or (1:6) [Zr_6_]:**H**_**2**_**TFS** stoichiometric ratio gave the two mixed-linker MOFs labeled as **PF-MOF1** and **PF-MOF2**. Their PXRD patterns are also shown in Figure 2, whereas their thermogravimetric (TG) profiles are shown in Figure 3. PSE did not seem to affect the structural integrity of the pristine fumarate; the diffractograms are nearly identical, and they clearly show that both PF-MOFs are isostructural to **MOF-801**. The only difference is in the intensity of a small peak at 2θ of ∼ 13° (already present in **MOF-801**) that slightly increases with the increasing amount of **H**_**2**_**TFS** used for the exchange. To gain additional information on the structural features of the presented PF-MOFs, an *ab initio* indexing using the N-TREOR suite^55^ was carried out. In all cases, cubic cells were found: *a* = 17.8469 Å, V = 5684 Å^3^ (**MOF-801**); *a* = 17.8498 Å, V = 5687 Å^3^ (**PF-MOF1**); *a* = 17.8621 Å, V = 5699 Å^3^ (**PF-MOF2**); *a* = 18.0690 Å, V = 5899 Å^3^ (**ZrTFS**). The results indicate that the unit cell size remains almost unchanged when passing from **MOF-801** to **PF-MOF1** to **PF-MOF2**, thus suggesting that very small differences exist between these three materials, whereas **ZrTFS** displays a larger unit cell. Integration of ^19^F and ^1^H NMR peaks of the digested samples allowed us to quantify the relative amounts of **FUM**^**2–**^, AA, and **TFS**^**2–**^ present in the lattice. In the framework structure of **MOF-801**, the externally added **TFS**^**2–**^ linkers may be incorporated through the replacement of either the pristine fumarates or the capping ligands (either acetates or water/hydroxide) placed in its defective sites, given the hypothesis that AA induced missing cluster defects.^56^ In the exchanged samples, after treatment with **H**_**2**_**TFS**, the signal of AA disappears. This suggests that the tetrafluorosuccinate replaces AA at defective sites in its monoprotonated form (**HTFS**^**–**^). Based on the combination of NMR analysis (Figures S6 and S7) and TGA (Figure 3), the proposed formulae for **PF-MOF1** and **PF-MOF2** are [Zr_6_O_4_(μ_3_-OH)_4_(μ_1_-OH)_2.08_(H_2_O)_2.08_(**FUM**)_4.04_(**HTFS**)_1.84_] and [Zr_6_O_4_(μ_3_-OH)_4_(μ_1_-OH)_1.83_(H_2_O)_1.83_(**FUM**)_4.04_(**HTFS**)_2.09_], respectively. Based on these formulae, we can conclude that no **TFS**^**2–**^/**FUM**^**2–**^ exchange has occurred and that **HTFS**^**–**^ has been grafted at defective sites, affording compounds with similar stoichiometry. Apparently, the fluorination degree is not appreciably increased by the use of more concentrated **H**_**2**_**TFS** solutions (1:2–1:6 **MOF-801**:**H**_**2**_**TFS** stoichiometric ratios) to foster a larger fluorinated linker uptake. TGA analysis of the four MOFs (Figure 3) shows a different thermal behavior depending on their fluorination extent. **MOF-801** contains almost 30 wt % of water, and it starts to decompose at temperatures higher than 380 °C. **ZrTFS** contains a smaller amount of solvent than **MOF-801** (about 7%), indicative of its lower porosity. In addition, the thermal stability is much lower (*T*_dec_ ≈ 260 °C), as expected for the lower Zr–O coordination bond strength of the (less basic) **TFS**^**2–**^ compared to that of **FUM**^**2–**^. The two PF-MOFs have a thermal behavior, which is intermediate between **ZrTFS** and **MOF-801**. The only difference is the water content, higher for **PF-MOF2** (23%, with a higher tetrafluorosuccinate loading) with respect to **PF-MOF1** (15%); the thermal stabilities though are comparable (*T*_dec_ ≈ 280 °C).

### Gas Adsorption Properties

#### N_2_ Adsorption

**Figure 4:**
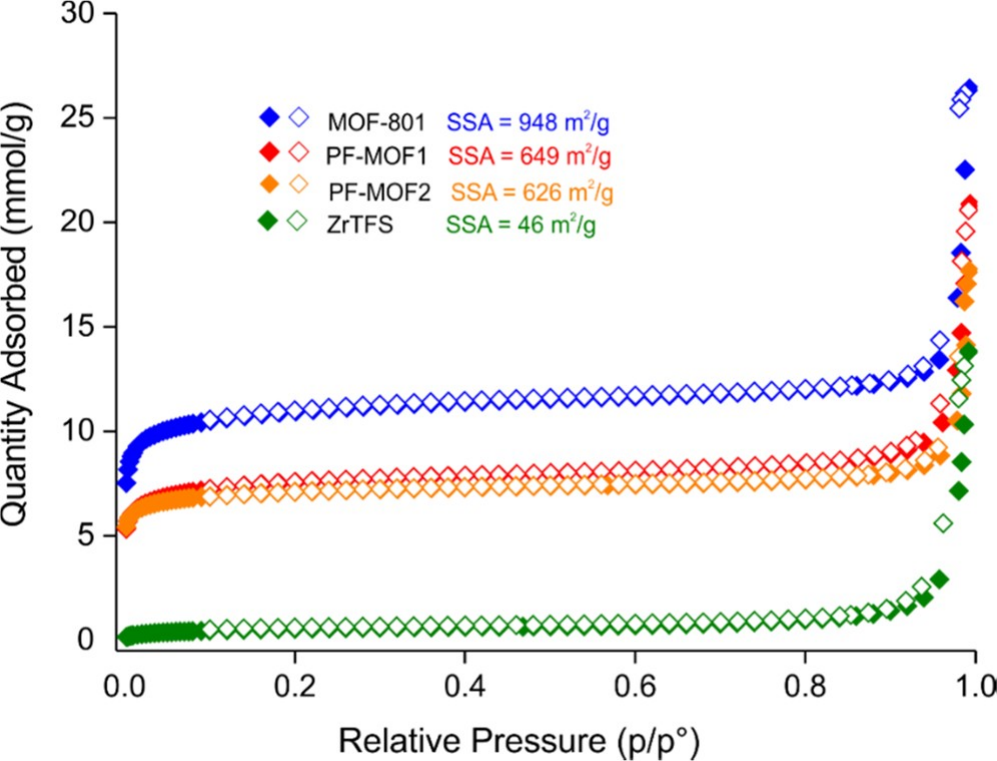
N_2_ adsorption isotherms measured at −196 °C on the four MOFs discussed in this work. Empty symbols denote desorption branches.

The porous nature of the four samples was investigated through N_2_ adsorption at −196 °C on desolvated samples. As shown in Figure 4, all compounds display type I isotherm, typical of microporous materials. **MOF-801** has the highest BET specific surface area (SSA) value (948 m^2^ g^–1^), and this result is in line with that obtained by other groups in the literature working with the same compound (833 m^2^ g^–1^ found by Serre and co-workers;^57^ 990 m^2^ g^–1^ reported by Yaghi and co-workers).^49^ The accessible surface area then decreases with an increasing content of the aliphatic fluorinated linker **TFS**^**2–**^, passing from 649 and 626 m^2^ g^**–**1^ for **PF-MOF1** and **PF-MOF2**, respectively, to only 46 m^2^ g^**–**1^ for **ZrTFS**. This drastic reduction of the surface area was also found in the hydrogenated counterpart of **ZrTFS**, zirconium succinate MIP-203-S.^58^ In there, it is claimed that this MOF does not show any accessible porosity to N_2_ at 77 K, presumably because after thermal activation under vacuum to remove the guest molecules, it tends to stay in a closely packed form that is not accessible to nitrogen. Even in the presence of bipyridyl-based auxiliary ligands, the framework empty volume is not accessible to N_2_, as observed in a family of cadmium succinate mixed-linker MOFs.^59^ Despite the different crystal topologies, it is reasonable to draw the same kind of conclusion for **ZrTFS** as well. This is further confirmed by the fact that the surface area of **ZrTFS** does not change when the equilibration time during the isotherm collection is doubled from 30 to 60 s. To gain additional insights into the textural properties of **ZrTFS**, the theoretical MOF surface area was estimated using a Monte Carlo procedure, which randomly places spheres with a given diameter in the free space (N_2_ to match experiments—sphere diameter = 3.681 Å)^60^ and calculates the interface between the spheres and the atoms (for further details and explanations, see the webpage: https://mausdin.github.io/MOFsite/mofPage.html). This is a commonly used procedure, and it normally gives reasonable results.^61^ By applying this procedure on **ZrTFS**, we have obtained a surface area of 226.8 m^2^ g^**–**1^. This value is reasonable if the pore size is taken into consideration, but it is much higher than the experimentally measured surface area. This discrepancy may be justified by a window size limitation for N_2_ free diffusion through the MOF pores. The micropore size distribution of the four samples retrieved from the NLDFT analysis (Figure S8) does not show a significant change when passing from the homolinker to the mixed-linker materials. The main contribution to the total pore volume comes from pores in the 10 ≤ *w* ≤ 12 Å range, in line with the Zr···Zr distance found in the crystal structure between adjacent [Zr_6_] nodes (∼13 Å).^49^ The percentage of the ultramicropore volume decreases with an increasing extent of fluorinated linker insertion, passing from 77% in **MOF-801** to 72 and 62% in **PF-MOF1** and **PF-MOF2**, respectively. Degradation coming from PSE cannot be considered the main cause of the observed microporosity reduction since the small amount of zirconium found in solution after the exchange indicates a very small degree of degradation (Table S2). This is also supported by the SEM analysis, which shows that the morphology and crystallite size seen in the parent **MOF-801** are preserved upon PSE (Figures S9–S11). Thus, PSE does not seem to affect the framework stability. The total pore volume derived from the N_2_ isotherm at −196 °C, measured at *p*/*p*_0_ = 0.98, equals 0.57, 0.45, 0.36, and 0.17 cm^3^ g^**–**1^ for **MOF-801**, **PF-MOF1**, **PF-MOF2**, and **ZrTFS**, respectively.

#### CO_2_ Adsorption

**Figure 5:**
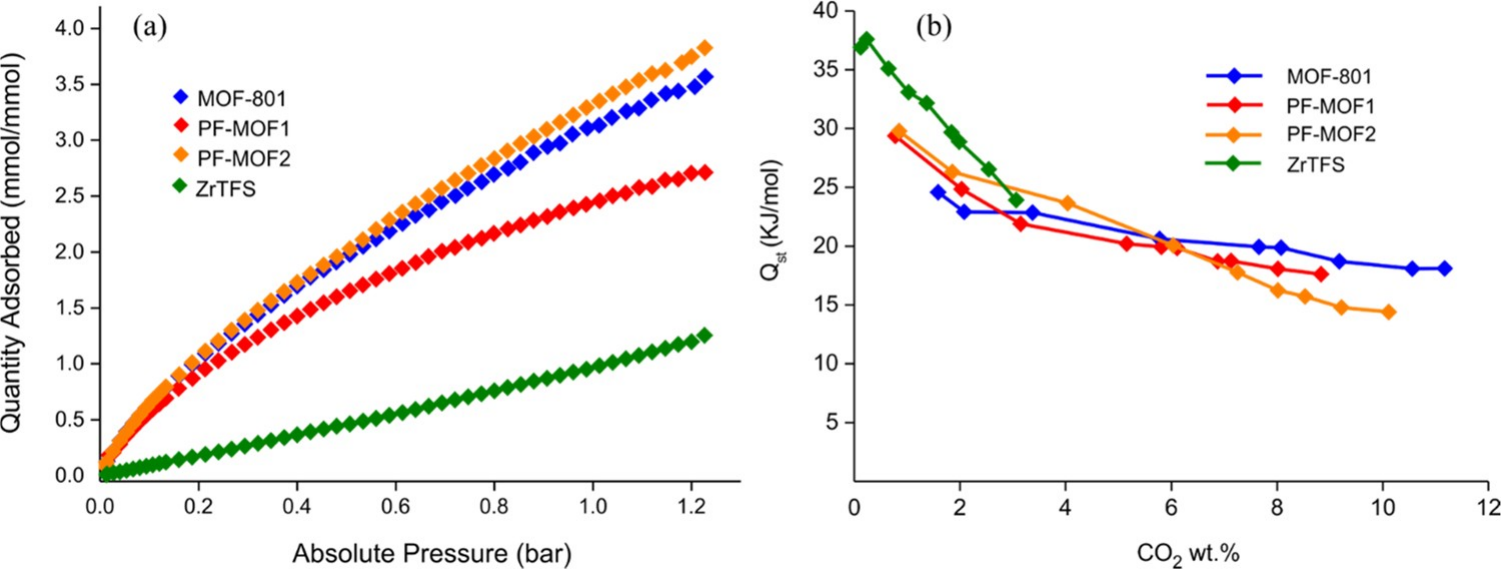
(a) CO_2_ adsorption isotherms measured at 25 °C on the four MOFs. (b) CO_2_ isosteric heat of adsorption as a function of surface coverage.

The four MOFs were tested as CO_2_ adsorbents at *T* = 0 and 25 °C and p_CO2_ up to 1.2 bar. The corresponding isotherms at an ambient temperature are reported in Figure 5a, where the amounts of CO_2_ adsorbed are reported as mmol(CO_2_) adsorbed per mmol (MOF) to account for the material density variation upon the fluorinated linker insertion. The more conventional mmol g^**–**1^ unit is reported in Table 1 and Figure S12. To quantify the strength of the host–guest interactions, the isosteric heat of adsorption (*Q*_st_) of CO_2_ was evaluated from the isotherms recorded at *T* = 0 and 25 °C, applying a variant of the Clausius–Clapeyron equation (Figure 5b). In order to validate the calculation made on two temperature points, for **ZrTFS** and **PF-MOF1***Q*_st_ of CO_2_ was also re-calculated using three temperatures (T = −20, 0 and 25 °C; Figures S13 and S20). An alternative approach for the extrapolation of the *Q*_st_ values at zero coverage is the virial fitting of the adsorption isotherms.^35^ The absolute values and the general trends calculated in our samples are identical to those obtained through the Clausius–Clapeyron equation (Figures S14–S18 and Tables S3–S6). The isosteric heat of adsorption reflects the interaction strength between CO_2_ and the inner pore walls of the MOFs. Finally, we estimated the CO_2_/N_2_ selectivity using the ratio of the initial slopes in the Henry region of the (CO_2_ and N_2_) adsorption isotherms measured at 25 °C (Figure S19). From the critical and comparative analysis of the results, we can state that the good performance in CO_2_ adsorption depends on two factors: BET area and fluorinated linker content. The former is predominant in **MOF-801**, where the absence of F atoms is compensated by the high SSA value. Consequently, **MOF-801** shows the best performance among the MOFs considered in this study in terms of CO_2_ loading on a gravimetric basis: 2.42 (10.6 wt %) and 3.51 (15.4 wt %) mmol g^**–**1^ at *T* = 25 and 0 °C, respectively. On the other hand, the introduction of **TFS**^**2–**^ partially reduces the accessible surface area (and the related CO_2_ loading) but considerably improves the thermodynamic affinity of the material for CO_2_ and its CO_2_/N_2_ selectivity. The latter is particularly important in the purification of postcombustion industrial flue gases, where the amount of CO_2_ is very low (4–30%). The high Henry and IAST selectivity achieved by **PF-MOF2** and **ZrTFS**, in particular, may be ascribed to the presence of fluorine-decorated ultramicropores that hamper N_2_ diffusion (because of its large kinetic diameter) but favor CO_2_ adsorption through the beneficial gas–fluorine interaction. On this basis, the best compromise can be found in the **PF-MOF2**, with a good CO_2_ loading of 2.1 mmol g^**–**1^ (9.3 wt %) at an ambient temperature and 1 bar, a *Q*_st_ value of almost 30 kJ mol^**–**1^, and the good CO_2_/N_2_ selectivity of 95 (Henry)/41 (IAST). The same conclusion can be drawn from the isotherms reported in Figure 5a, where **PF-MOF2** shows the highest CO_2_ uptake: 3.7 mmol_CO2_ mmol_MOF_^**–**1^ at *p* = 1 bar. In comparison with other PF-MOFs reported in the literature (Table S7), at ambient (T,p) conditions (25 °C, 1 bar), **PF-MOF2** outperforms F4-UiO-66(Ce) (1.5 mmol g^–1^ at 293 K),^21^ but it is less efficient than F4-MIL-140A(Ce) (2.4 mmol g^–1^)^25^ and AlFFIVE-1-Ni/NbOFFIVE-1-Ni (2.7/2.2 mmol g^–1^, respectively) published by the team of Eddaoudi in 2016^23,24,62^ or than SIFSIX-18-Ni (3.0 mmol g^–1^), reported by Zaworotko and co-workers in 2019.^20^

**Table 1:** Main CO_2_ Adsorption Data for the Four MOFs in This Study

					CO_2_ quantity adsorbed (*p* = 1 bar) [mmol g^–1^]	
	BET area [m^2^ g^–1^]	*Q*_st_ [kJ mol^–1^]	CO_2_/N_2_ selectivity (Henry)	CO_2_/N_2_ selectivity (IAST)	*T* = 25 °C	*T* = 0 °C
**MOF-801**	948	24.6	23	25	2.42 (10.6 wt %)	3.51 (15.4 wt %)
**PF-MOF1**	649	29.4	30	34	1.58 (6.9 wt %)	2.22 (9.8 wt %)
**PF-MOF2**	626	29.8	95	41	2.10 (9.3 wt %)	2.78 (12.2 wt %)
**Zr_TFS**	46	37.6	∞^a^	∞^a^	0.56 (2.5 wt %)	1.24 (5.4 wt %)

a The N_2_ adsorption in **ZrTFS** at *T* = 25 °C is practically zero at low coverage.

### DFT Study of the CO_2_–Framework Interaction

**Figure 6:**
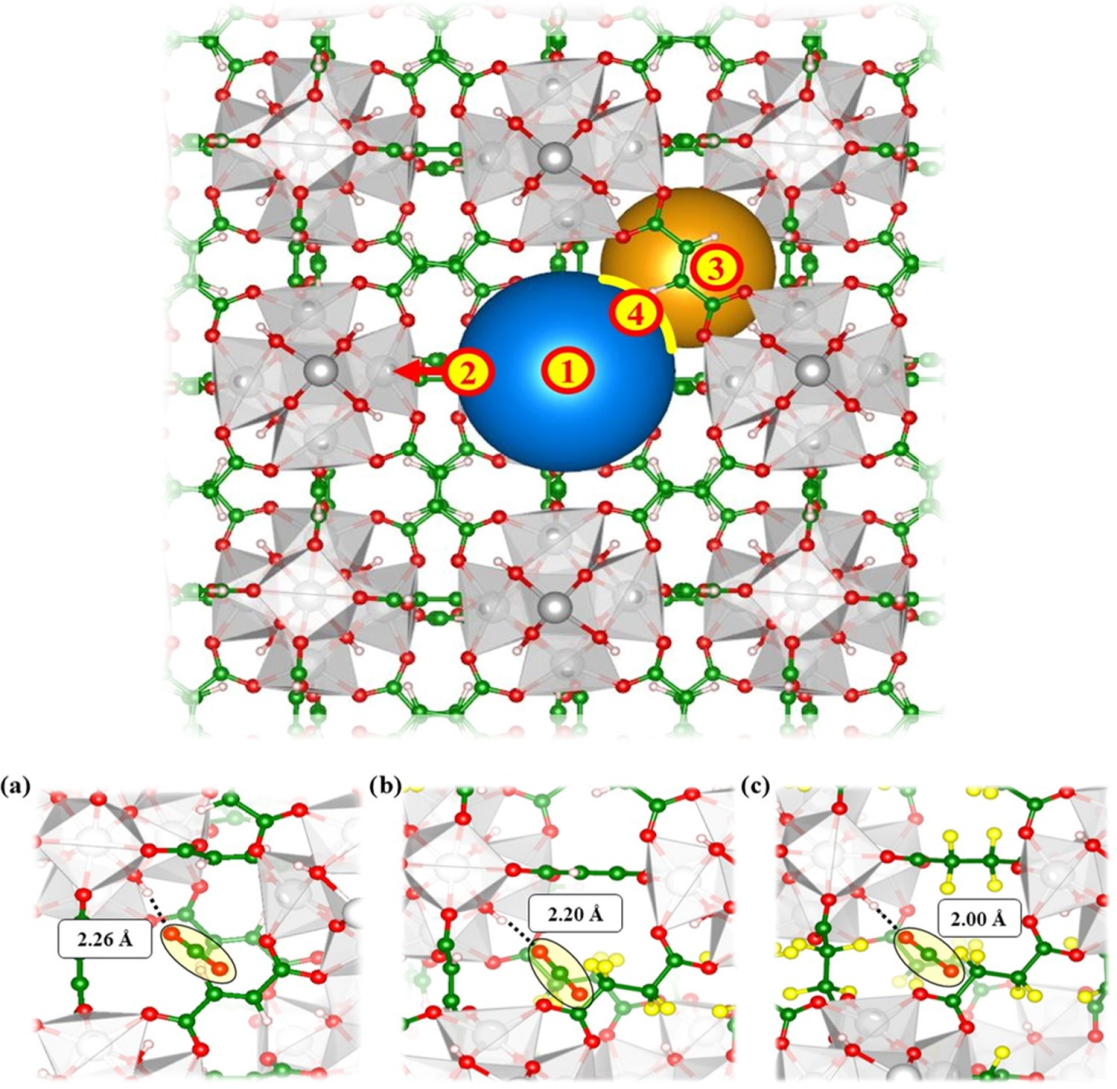
Top: optimized DFT structure of **ZrTFS**. The blue and the orange spheres represent the octahedral and tetragonal pores, respectively; the yellow curved line represents the window between the two pores; the numbers indicate the different sites taken into account for the evaluation of CO_2_ adsorption enthalpies (Δ*E*_ads_) in reference to Table 2. Bottom: detail of the hydrogen bonds between CO_2_ and the OH group for (a) **MOF-801**, (b) **PF-MOF**, and (c) **ZrTFS**.

To gain insight into the effect of the fluorinated ligand on the MOF structural properties and CO_2_ adsorption ability, DFT simulations in the gas phase at the [PBE/MOLOPT] level of theory were carried out. As a first step in the computational analysis, the structures of **MOF-801** and **ZrTFS** were optimized. For **MOF-801**, the starting geometry was the defect-free crystal lattice with formula [Zr_6_O_4_(OH)_4_(**FUM**)_6_], while for **ZrTFS** the initial guess [Zr_6_O_4_(OH)_4_(**TFS**)_6_] was considered. To evaluate the effect of the presence of both linkers in the same solid phase on the CO_2_ adsorption performance from a theoretical viewpoint, a hypothetical (simplified) structure of minimal formula [Zr_6_O_4_(OH)_4_(**FUM**)_4_(**TFS**)_2_] (**PF-MOF**) was also built *in silico* and subsequently optimized. Albeit this computational model cannot be considered fully representative of the real exchanged materials **PF-MOF1** and **PF-MOF2**, it is a useful reference for a comparison between a mixed-linker MOF and its homolinker counterparts. Indeed, the design of computationally representative defective structures is too challenging, as the linker is placed onto the defects randomly. On these models, a joint variable-cell and atomic position optimization was performed (see the Computational Details section). As shown in Table 2, the optimized lattice parameters are in perfect agreement with the experimentally determined values, with the reproduction of the slight unit cell size increment moving from pure fumarate to the fluorinated systems. These MOFs feature tetrahedral and octahedral pores interconnected by triangular windows, with a lattice structure similar to that of UiO-66.^63^ The windows are smaller than the pores (Figure 6 and Table 2), and they could be responsible for the percolation of the gas inside the MOF. The gas adsorption process is driven by two main factors: the host–guest chemical affinity and the (high) accessible SSA. Another interesting factor that deeply influences the adsorption performance is the gas percolation and diffusion inside the material.^64^ Literature evidence suggests that MOFs featuring high CO_2_ chemical affinity but with very small pore windows are poor adsorbents.^65,66^ To compare the window and pores of the different models, the surface of the window is defined as the area of the triangle defined by the three hydrogen (or fluorine) atoms, as indicated in Figure S21a, while the pore dimension is evaluated as the distance between opposite hydrogen or fluorine atoms in Figure S21b. The average values of the cavities and window size are summarized in Table 2. Moving from **MOF-801** to **ZrTFS**, the octahedral pore size does not change significantly, while the window area and the tetrahedral pore size are considerably reduced. Moreover, the mixed-linker **PF-MOF** shows a variable pore width (between 6 and 9 Å; Table 2) that depends on the presence of either **TFS**^**2–**^ or **FUM**^**2–**^. To gain deeper insights into the preferential CO_2_ adsorption sites in these materials, CO_2_ was introduced in the computational model and located in four different lattice positions (Figure 6): in the center of the octahedral cavity (1), close to the zirconium ions (2), in the center of the tetrahedral pore (3), and in the middle of the pore windows (4). Afterward, the ensemble was reoptimized. The CO_2_ adsorption energy (Δ*E*_ads_) is then calculated as the energy difference between the optimized [MOF + CO_2_] ensemble and the separated components; a negative value indicates a favorable interaction, while the opposite holds for positive Δ*E*_ads_ values. The most favorable adsorption site is the tetrahedral pore, even if it is slightly smaller than the octahedral one. This energy stabilization comes from a hydrogen bond interaction between CO_2_ and a hydroxyl group on the inorganic cluster (Figure 6), which is known to be the most favorable adsorption site for polar and quadrupolar species in UiO-66.^67^ Apparently, there is no simple correlation between F-functionalization and MOF–CO_2_ interaction. We can only state that the interaction of CO_2_ with the linker F atoms is not particularly strong, at least at the computational level used here. To better understand the role of fluorine atoms in increasing the affinity with CO_2_, the Mulliken partial charges on F species were evaluated as already reported in the literature for the SIFSIX family MOFs.^68^ In particular, the SiF_6_^2–^ anion was taken as the benchmark model, obtaining a partial F charge of −0.76e, in agreement with the values found for similar systems.^68^ Subsequently, the partial charge on the fluorine atoms of the **H**_**2**_**TFS** linker was calculated at the same level of theory, obtaining an average value of −0.25e. This clearly indicates a reduced C–F bond polarization in **H**_**2**_**TFS** if compared to the Si–F bond polarization in SiF_6_^2–^. Consequently, these results suggest a lower CO_2_ affinity of **ZrTFS** with respect to a general SIFSIX system. If the (Δ*E*_ads_)_T_ values found for the different materials are compared, an increase in the interaction energy is found when moving from **MOF-801** to **ZrTFS**, in perfect agreement with the *Q*_st_ trend reported in Table 1. It is to be noted that the window site in **ZrTFS** is associated with a positive (Δ*E*_ads_) value, which suggests that CO_2_ diffusion through the windows is unfavorable, explaining the low adsorption capacity of this MOF.

**Table 2:** Main Calculated Structural Parameters and CO_2_ Adsorption Enthalpies for **MOF-801**, **PF-MOF**, and **ZrTFS**

	structural parameters			CO_2_ Δ*E*_ads_ (eV)			
	cell (Å)	window area (Å^2^)	pore (Å)^a^	pore Oct. center (1)	pore Zr (2)	pore Tetr. center (3)	window (4)
**MOF-801**	18.00	9.3	9.6/8.6	–0.19	–0.29	–0.34	–0.29
**PF-MOF**	18.04	9.1	10.0/6.3–9.0	–0.18	–0.27	–0.47	–0.29
**ZrTFS**	18.17	5.0	10.1/6.6	–0.18	–0.31	–0.49	0.32

a We report both octahedral and tetrahedral pore sizes, respectively.

## Conclusions

The novel perfluorinated MOF **ZrTFS** containing tetrafluorosuccinic acid and isostructural to **MOF-801** has been synthesized and its crystal structure was solved and refined from PXRD data. The presence of fluorine atoms on the alkyl chains hinders gas adsorption; consequently, the measured surface area of this MOF is very low. To obtain a fluorinated material with an acceptable surface area, **MOF-801** was taken as the starting point to carry out PSE in aqueous solutions with variable amounts of **H**_**2**_**TFS**. In the two exchanged materials, the amount of a fluorinated linker is about the same despite the different concentrations used in the synthesis, and it is most likely placed in the precursor defective sites. This suggests that the postsynthetic exchange involves only acetate and not fumarate. The as-obtained PF-MOFs show lower CO_2_ adsorption capacity with respect to **MOF-801** (most likely because of the lower surface area) but higher CO_2_/N_2_ selectivity and CO_2_ heat of adsorption. These parameters are directly proportional to the extent of a fluorinated linker present in the lattice, proving the beneficial effect of the presence of fluorinated groups on the CO_2_ affinity of the resulting material. DFT-optimized structures of **ZrTFS** account for the low diffusion of gases into the framework due to the reduction of the window size. The theoretical model of [CO_2_@PF-MOF] ensembles shows the formation of strong hydrogen-bonding interactions between CO_2_ and hydroxyl groups of the [Zr_6_] clusters dangling in the tetrahedral cavities and favored by the exposed fluorine atoms. Although a real linker exchange did not occur in the present work, being a defect functionalization, the PSE methodology is very efficient for the preparation of mixed-linker MOFs with tunable properties and opens new horizons for selected applications such as gas mixture separation and purification. Current work is undergoing in our laboratories in this direction.

## Supporting Information Available

IR spectra, PXRD patterns of **ZrTFS** obtained through different synthetic methods, PXRD structure determination of **ZrTFS**, its Rietveld refinement plot and crystallographic details, ^1^H NMR and ^19^F NMR spectra of the digested samples of **MOF-801**, **PF-MOF1**, and **PF-MOF2** and related linker quantification, ICP-OES analysis of the reaction supernatant after PSE on **MOF-801**, MOF pore size distribution, calculation of the CO_2_/N_2_ Henry selectivity, CO_2_ adsorption isotherm plot in [mmol g^**–**1^] units at 25 °C, further *Qst* calculations using three temperatures and geometrical considerations on the DFT-optimized structures (PDF)

## Accession Codes

CCDC-2160377 contains the crystallographic data for ZrTFS that can be obtained free of charge from the Cambridge Crystallographic Data Centre (http://www.ccdc.cam.ac.uk/data_request/cif).
