# Supplementary material for: Increased CO2 Affinity and Adsorption Selectivity in MOF-801 Fluorinated Analogues
Source: ACS Appl Mater Interfaces. 2022 Aug 30;14(36):40801–11. doi: 10.1021/acsami.2c07640 (PMC9478941; doi:10.1021/acsami.2c07640)
Supplement: Supplementary file 1 — am2c07640_si_001.pdf [file am2c07640_si_001.pdf]

Supporting Information

Increased CO_2 Affinity and Adsorption Selectivity In MOF-801 Fluorinated Analogues

Diletta Morelli Venturi,[a] Maria Sole Notari,[a] Roberto Bondi,[a] Edoardo Mosconi,[b] Waldemar Kaiser,[b] Giorgio Mercuri,[c,d] Giuliano Giambastiani,[c] Andrea Rossin,[,c] Marco Taddei*[,e] and Ferdinando Costantino*[,a]*

[a] Department of Chemistry, Biology and Biotechnology, Università degli Studi di Perugia, via Elce di Sotto, 8 - 06123 Perugia, Italy. E-mail: ferdinando.costantino@unipg.it

[b] Computational Laboratory for Hybrid/Organic Photovoltaics (CLHYO), Istituto CNR di Scienze e Tecnologie Chimiche "Giulio Natta" (CNR-SCITEC), Via Elce di Sotto 8, 06123, Perugia, Italy

[c] Istituto di Chimica dei Composti Organometallici (CNR-ICCOM), Via Madonna del Piano 10, 50019 Sesto Fiorentino (Firenze), Italy. E-mail: a.rossin@iccom.cnr.it

[d] Scuola del Farmaco e dei Prodotti della Salute, Università di Camerino, Via S. Agostino 1, 62032 Camerino, Italy.

[e] Department of Chemistry and Industrial Chemistry, University of Pisa, Via Giuseppe Moruzzi 13, 56124, Pisa, Italy. E-mail: marco.taddei@unipi.it

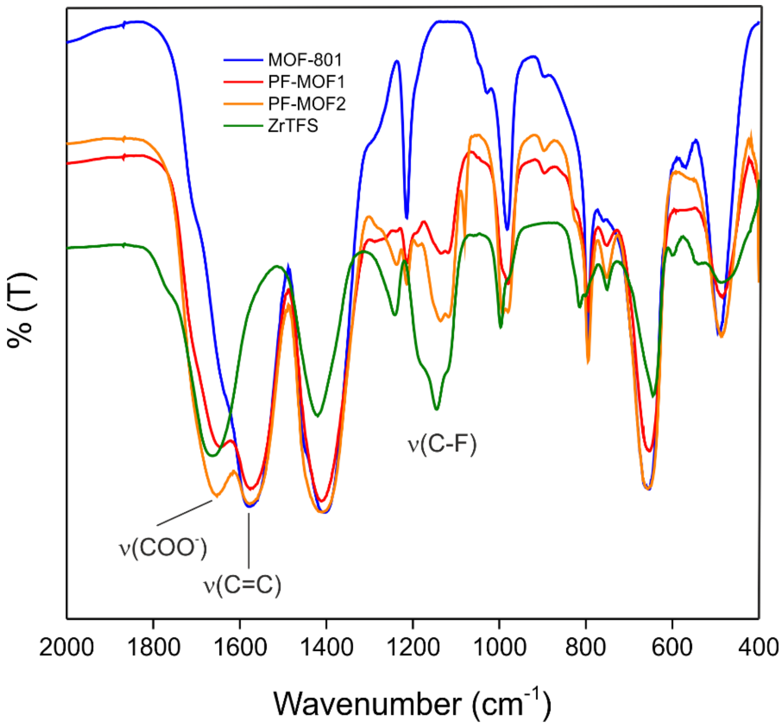

Figure S1. IR spectra (KBr pellet) in the 2000-400 cm^{-1} region of the four MOFs described in this study.

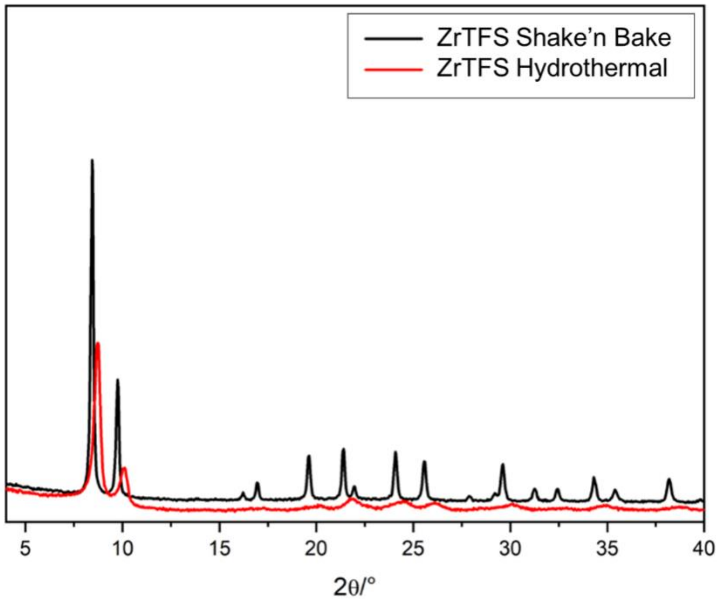

Figure S2. PXRD patterns of **ZrTFS** obtained through a classical hydrothermal synthesis (red line) or through the "Shake 'n Bake" methodology (black line).

Powder X-ray Diffraction Structure Determination of ZrTFS

Indexing of the diffraction patterns of **ZrTFS** was performed with *TOPAS* (v. 4.2).[1] The analysis of systematic extinctions for space group assignment was performed using the *Chekcell* program.[2] Structure solution was performed *ab initio* using the software *FOX 4.*[3] Initially, [ZrO_8] cubes were employed to identify the location of the inorganic clusters. Then, the position of one Zr atom and two O atoms (corresponding to μ_3-O and μ_3-OH) was fixed and two independent **TFS** fragments were included in the asymmetric unit. Bond lengths and bond angles were restrained to correctly reproduce the geometry of the linkers. The centers of mass of these fragments were placed on special positions with coordinates (0.5, 0.25, 0.25) and (0.25, 0, 0.75), respectively. Two Ne atoms, whose occupancy was left free to refine, were also used to mimic adsorbed water molecules. A set of anti-bump restraints was employed to prevent unrealistically short intermolecular contacts: Zr-F = 4.5 Å; Zr-Ne = 4.0 Å; O-Ne = 2.0 Å; C-Ne = 2.0 Å; F-Ne = 2.0 Å. The initial model was then refined using the Rietveld method in *TOPAS* (v. 4.2).[1] First, a Pawley refinement was carried out to model background, sample displacement, profile shape parameters (Full_Axial_Model, CS_L, CS_G) and lattice parameters (Cubic). Then, Rietveld refinement was performed to model the atomic coordinates and atomic S3 displacement parameters. In order to maintain the mass center of the linker in special position (*i.e.* rotation of the alkyl chain outside of the plane defined by carboxylic groups), the alkyl chains, defined by C11, C12, O11, O12, F11, F12 atoms and C21, C22, O21, O22, F21, F22 were modelled by employing two Z-matrix. In order to describe the geometry of the alkyl chain correctly, a dummy atom (whose occupancy factor was set to zero) was employed in the Z-matrix: X1 was placed in the middle between the C11 and the C12 of alkyl chain in special position (0.5, 0.25, 0.25) whereas X2 was placed in the middle between C21 and C22 in special position (0.25, 0, 0.75). Rotational parameters were left free to refine. All the other

atoms were refined individually, using eight distance restraints and two angle restraints. The atomic displacement parameters for Zr were refined independently with the oxygen of the cluster (muO1, muO2), while those of the light atoms were constrained to the same value. The Ne atoms used to mimic water molecules in the pores were converted to O atoms, whose occupancy factors were left free to refine. At the end of the refinement, all the parameters were refined together until convergence.

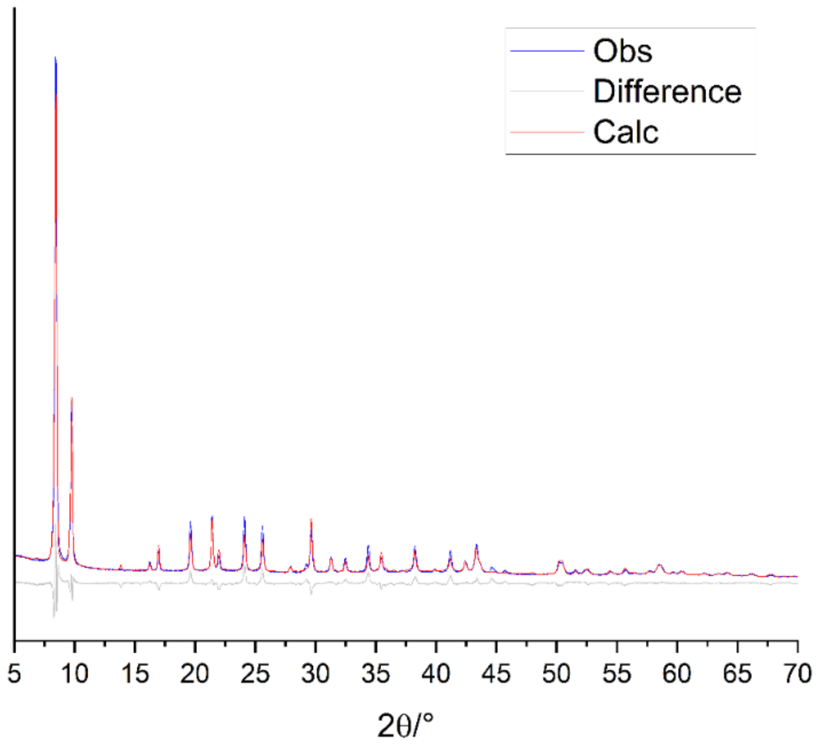

Figure S3. Rietveld refinement carried out on the PXRD pattern (λ= 1.5401 Å) of **ZrTFS** (synthetized *via* "shake and bake" methodology).

Table S1. Main crystallographic data and structure refinement details for **ZrTFS**.

Formula	$C_{24}H_4O_{32}F_{24}Zr_6$	$[Zr_6O_4(OH)_4(C_4O_4F_4)_6]$
M [g mol^{-1}]	1807.57	
Wavelength [Å]	1.54056	
T [°C]	25	
Crystal system	Cubic	
Space group	*Pn-3*	
Z	24	
a [Å]	18.081	
V [Å^3]	5910	
D_{calc} [g cm^{-3}]	2.212	
R_p	7.055	
R_{wp}	9.699	
R_F2	5.484	
GoF	5.23	

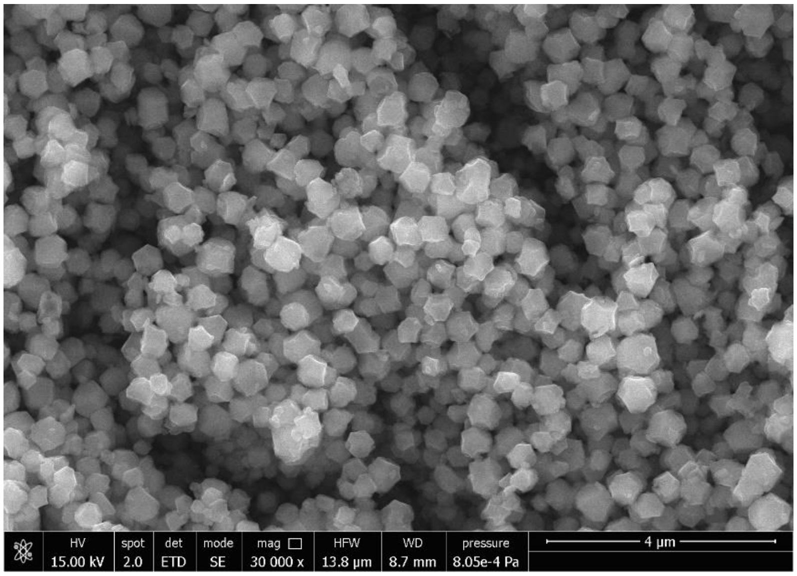

Figure S4. SEM image of **ZrTFS**.

^{1}H-NMR and ^{19}F-NMR spectra and related linkers quantification in MOF-801 and mixed-linker MOFs

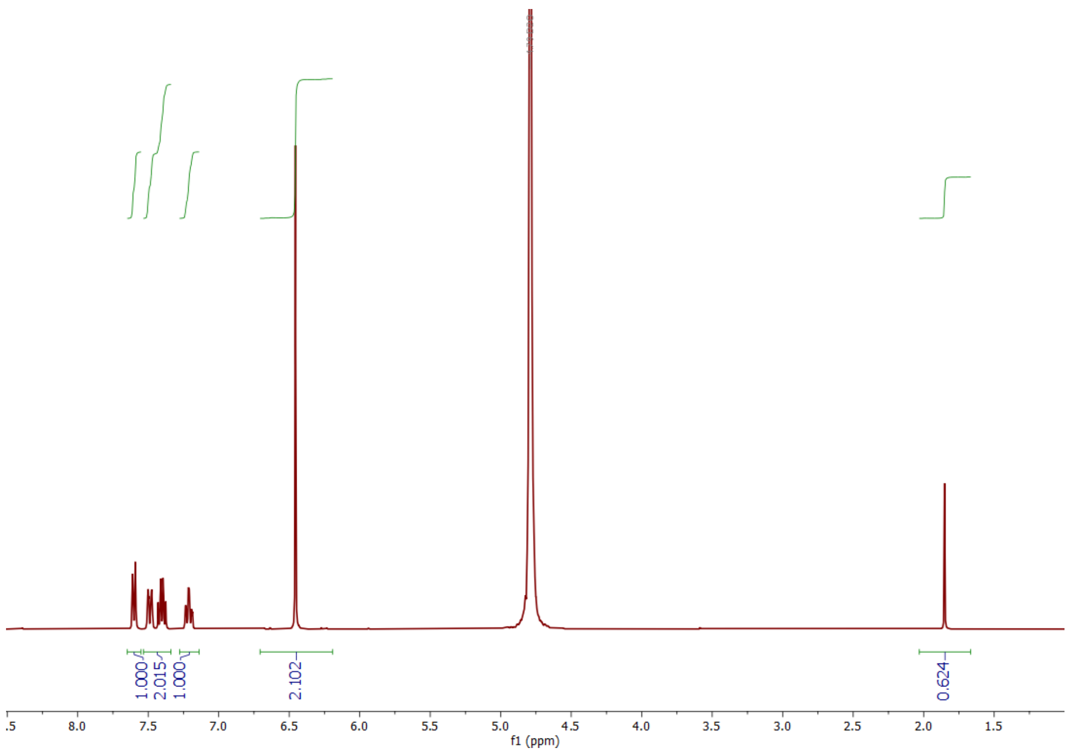

Figure S5. ^{1}H-NMR spectrum of **MOF-801**.

14.7 mg of desolvated sample was digested. The signal of **FUM^{2-}** falls at δ_H = 6.45 ppm and accounts for two protons. The signal of AA falls at δ_H = 1.85 ppm and accounts for three protons. The four signals of the internal standard 3-fluorobenzoic acid (3FBA) fall between δ_H = 7.16 and 7.64 ppm and each one accounts for one proton. Thus, to have a quantitative comparison of the two species, the integral of **FUM^{2-}** (2.102) must be divided by two, giving 1.051, whereas the integral of AA (0.624) must be divided by three, giving 0.208. The **FUM^{2-}**/AA ratio is therefore 5.05. Assuming the general formula [$Zr_6O_4(OH)_4$(**FUM**)$_{6-x}$(AA)$_{2x}$], the following can be written:

$$\frac{FUM^{2-}}{AA} = \frac{6-x}{2x} = 5.05$$

This leads to calculate a value of 0.54 for x, leading to the following proposed formula: $[Zr_6O_4(OH)_4(\mathbf{FUM})_{5.46}(AA)_{1.08}]$. Such a formula leads to calculate expected wt. % of 45.6% for $\mathbf{FUM^{2-}}$ and of 4.7% for AA. The absolute concentration of $\mathbf{FUM^{2-}}$ and AA in solution can be derived by dividing each integral by the value of the integral of 3FBA (1.000) and multiplying the result by its concentration (0.029 M), obtaining 0.030 M for $\mathbf{FUM^{2-}}$ and 0.006 M for AA. The wt. % of $\mathbf{FUM^{2-}}$ and AA can be derived by multiplying these values by the volume of the solution (1.5 mL) and the molecular weight of the respective anions (114 g/mol for $\mathbf{FUM^{2-}}$, 59 g/mol for AA) and dividing the obtained values (5.21 mg for $\mathbf{FUM^{2-}}$, 0.53 mg for AA) by the mass of the desolvated MOF digested (14.7 mg), resulting in 35.4% for $\mathbf{FUM^{2-}}$ and 3.6% for AA. The lower experimental wt. % obtained when compared to those expected from the formula proposed above suggests that the proposed formula overestimates the amount of $\mathbf{FUM^{2-}}$ and AA in the MOF. Given that the synthesis was carried out in aqueous medium, we can propose an alternative formula, where part of $\mathbf{FUM^{2-}}$ is also replaced by a *hydroxide/water couple*: $[Zr_6O_4(\mu_3\text{-}OH)_4(\mu_1\text{-}OH)_{2y}(H_2O)_{2y}(\mathbf{FUM})_{6-x-y}(AA)_{2x}]$. The values of x and y can be derived by solving the following equations:

$$\frac{FUM^{2-}}{AA} = \frac{6-x-y}{2x} = 5.05$$

$$\frac{m(FUM^{2-})}{m(MOF)} = \frac{(6-x-y)\times 114}{(91\times 6)+(16\times 4)+(4+2y)\times 17+(2y\times 18)+(6-x-y)\times 114+(2x\times 59)} = 0.354$$

The second equation imposes that the weight % of $\mathbf{FUM^{2-}}$ be equal to the experimental value obtained above. The values of x and y derived by solving these equations are 0.40 and 1.56, respectively, leading to the following proposed formula: $[Zr_6O_4(\mu_3\text{-}OH)_4(\mu_1\text{-}OH)_{3.12}(H_2O)_{3.12}(\mathbf{FUM})_{4.04}(AA)_{0.80}]$. To confirm the reliability of this formula, we refer to the TG curve displayed in Figure 3 in the main text, which shows that the MOF loses 30 wt% upon

desolvation (at 300 °C) and a further 30 wt% upon degradation of the organic part of the framework (at 700 °C), when only ZrO_2 is left. Thus, a ratio of 0.57 can be calculated between the mass% of leftover ZrO_2 (40%) and that of the desolvated MOF (70%). Given that the formula [$Zr_6O_4(\mu_3$-$OH)_4(\mu_1$-$OH)_{3.12}(H_2O)_{3.12}$(**FUM**)$_{4.04}$(AA)$_{0.80}$] has a FW of 1295 g/mol, and that 6 moles of ZrO_2 per mole of MOF are expected to remain after full decomposition, a ratio of 0.57 is calculated between the expected mass of leftover ZrO_2 and that of the desolvated MOF with the above formula, confirming that the proposed formula is in line with the experimental outcomes from the thermogravimetric analysis.

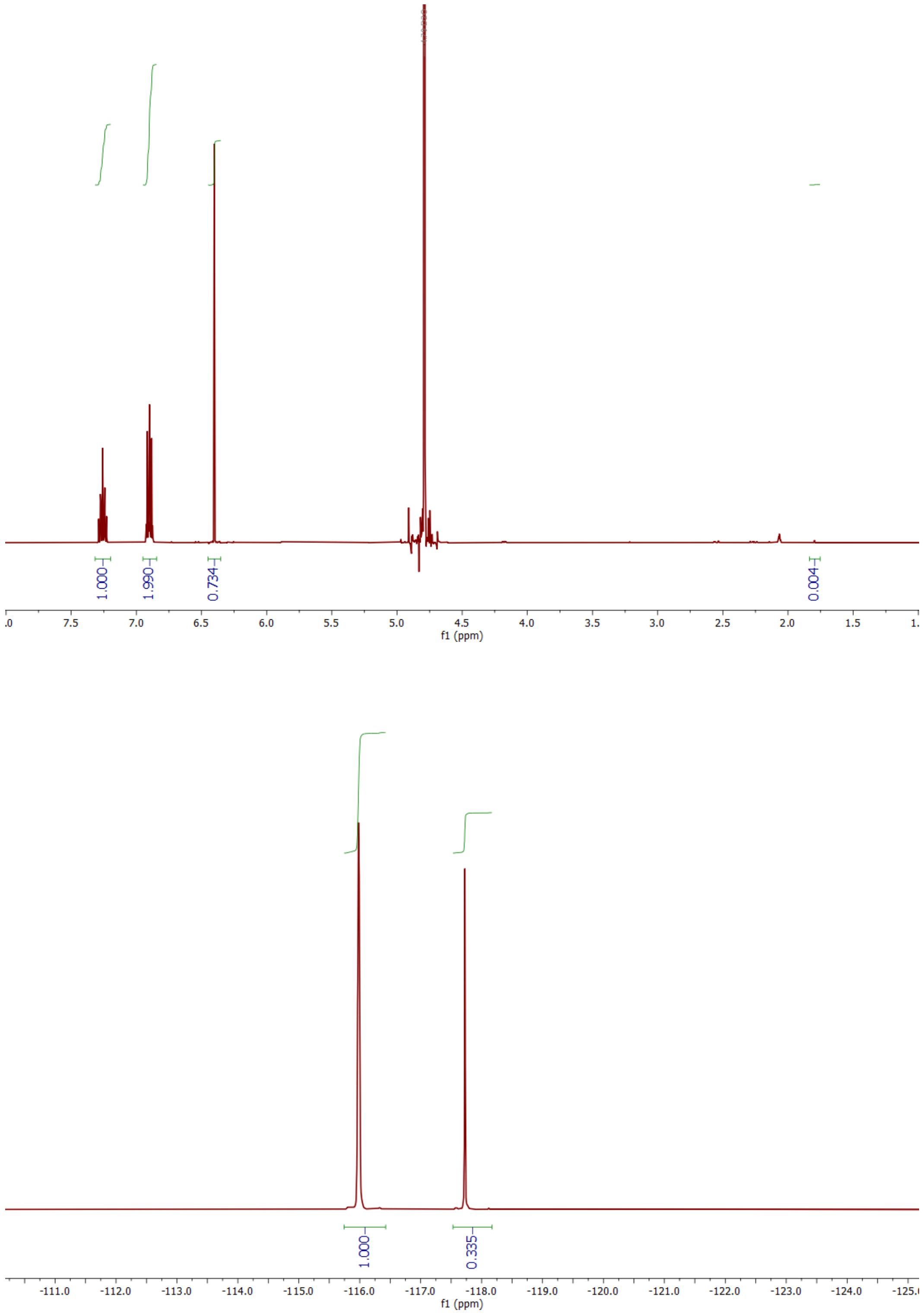

Figure S6. ^{1}H-NMR (top) and ^{19}F-NMR (bottom) spectra of **PF-MOF1**.

12.9 mg of desolvated sample was digested. In the ^{1}H-NMR spectrum, the signal of **FUM^{2-}** falls at δ_H = 6.40 ppm and accounts for two protons. The signal of AA falls at δ_H = 1.79 ppm and accounts for three protons. Thus, to have a quantitative comparison of the two species, the integral of **FUM^{2-}** (0.734) must be divided by two, giving 0.367, whereas the integral of AA (0.004) must be divided for three, giving 0.001. The **FUM^{2-}**/AA ratio is therefore 367, suggesting that practically no AA is present in the MIXMOF after PSE. The two signals of 2,6-difluorobenzoic acid (DFBA) fall at δ_H = 6.90 ppm (accounts for two protons) and δ_H = 7.26 ppm (accounts for one proton). Taking the value of the DFBA signal falling at 7.26 ppm (1.00), a **FUM^{2-}**/DFBA ratio of 0.367 is calculated. In the ^{19}F-NMR spectrum, the signal of **TFS^{2-}** falls at δ_F = −117.8 ppm and accounts for four fluorine atoms. The signal of DFBA falls at δ_H = −116.0 ppm and accounts for two fluorine atoms. Thus, to have a quantitative comparison of the two species, the integral of **TFS^{2-}** (0.335) must be divided by four, giving 0.084, whereas the integral of DFBA (1.000) must be divided by two, giving 0.500. A **TFS^{2-}**/DFBA ratio of 0.168 is thus obtained. Using the values of **FUM^{2-}**/DFBA and **TFS^{2-}**/DFBA ratios, a **FUM^{2-}**/**TFS^{2-}** ratio of 2.19 is obtained. Given the possibility that TFS could be present both in fully deprotonated **TFS^{2-}** form (installed in the framework in place of **FUM^{2-}**) and in monoprotonated **HTFS^{-}** form (installed at defective sites in place of AA or OH/H_2O), and assuming that the number of defects stays constant upon PSE, several scenarios can be envisioned, each leading to a different hypothetical formula for **PF-MOF1**. For the sake of simplicity, we report here only the result that best fits with the experimental data, *i.e.* exchange of **TFS^{2-}** *only at the defective sites*, with no incorporation in the framework replacing **FUM^{2-}**. Consequently, a general formula $[Zr_6O_4(\mu_3\text{-OH})_4(\mu_1\text{-OH})_x(H_2O)_x(\mathbf{FUM})_{4.04}(\mathbf{HTFS})_{3.92-x}]$ is proposed. The following equation can be written:

$$\frac{4.04}{3.92 - x} = 2.19$$

This leads to x = 2.08, and to the formula: $[Zr_6O_4(\mu_3\text{-}OH)_4(\mu_1\text{-}OH)_{2.08}(H_2O)_{2.08}$(**FUM**)$_{4.04}$(**HTFS**)$_{1.84}$]. Such a formula leads to calculate expected wt. % of 29.5% for **FUM**$^{2-}$ and of 22.3% for **HTFS**$^{-}$. The absolute concentration of **FUM**$^{2-}$ and **TFS**$^{2-}$ (note that the solution is strongly alkaline, so that **H$_2$TFS** is fully deprotonated) in solution can be derived by multiplying the **FUM**$^{2-}$/DFBA and **TFS**$^{2-}$/DFBA ratios by 0.1, obtaining 0.0367 M for **FUM**$^{2-}$ and 0.0168 M for **TFS**$^{2-}$. The wt. % of **FUM**$^{2-}$ and **HTFS**$^{-}$ in the MOF can be derived by multiplying these values by 1.0 (volume of the solution) and the molecular weight of the respective anions (114 for **FUM**$^{2-}$, 189 for **HTFS**$^{-}$) and dividing the obtained values (4.18 mg for **FUM**$^{2-}$, 3.18 mg for **HTFS**$^{-}$) by the mass of the desolvated MOF digested (12.9 mg), resulting in 32.4% for **FUM**$^{2-}$ and 24.7% for **HTFS**$^{-}$. These values are slightly higher than those expected from the formula proposed above. However, other scenarios where **TFS**$^{2-}$ substitutes for **FUM**$^{2-}$ in the framework lead to much larger discrepancies, suggesting that the proposed formula is the most reliable. To further confirm the reliability of this formula, we refer to the TG curve displayed in Figure 3 in the main text, which shows that **PFMOF-1** loses 18 wt% upon desolvation (at 280 °C) and a further 42 wt% upon degradation of the organic part of the framework (at 700 °C), when only ZrO_2 is left. Thus, a ratio of 0.49 can be calculated between the mass% of leftover ZrO_2 (40%) and that of the desolvated MOF (82%). Given that the formula $[Zr_6O_4(\mu_3\text{-}OH)_4(\mu_1\text{-}OH)_{2.08}$ $(H_2O)_{2.08}$(**FUM**)$_{4.04}$(**HTFS**)$_{1.84}$] has a FW of 1559 g/mol, and that 6 moles of ZrO_2 per mole of MOF are expected to remain after full decomposition, a ratio of 0.47 is calculated between the expected mass of leftover ZrO_2 and that of the desolvated MOF with the above formula, confirming that the proposed formula is in line with the experimental outcomes from the thermogravimetric analysis.

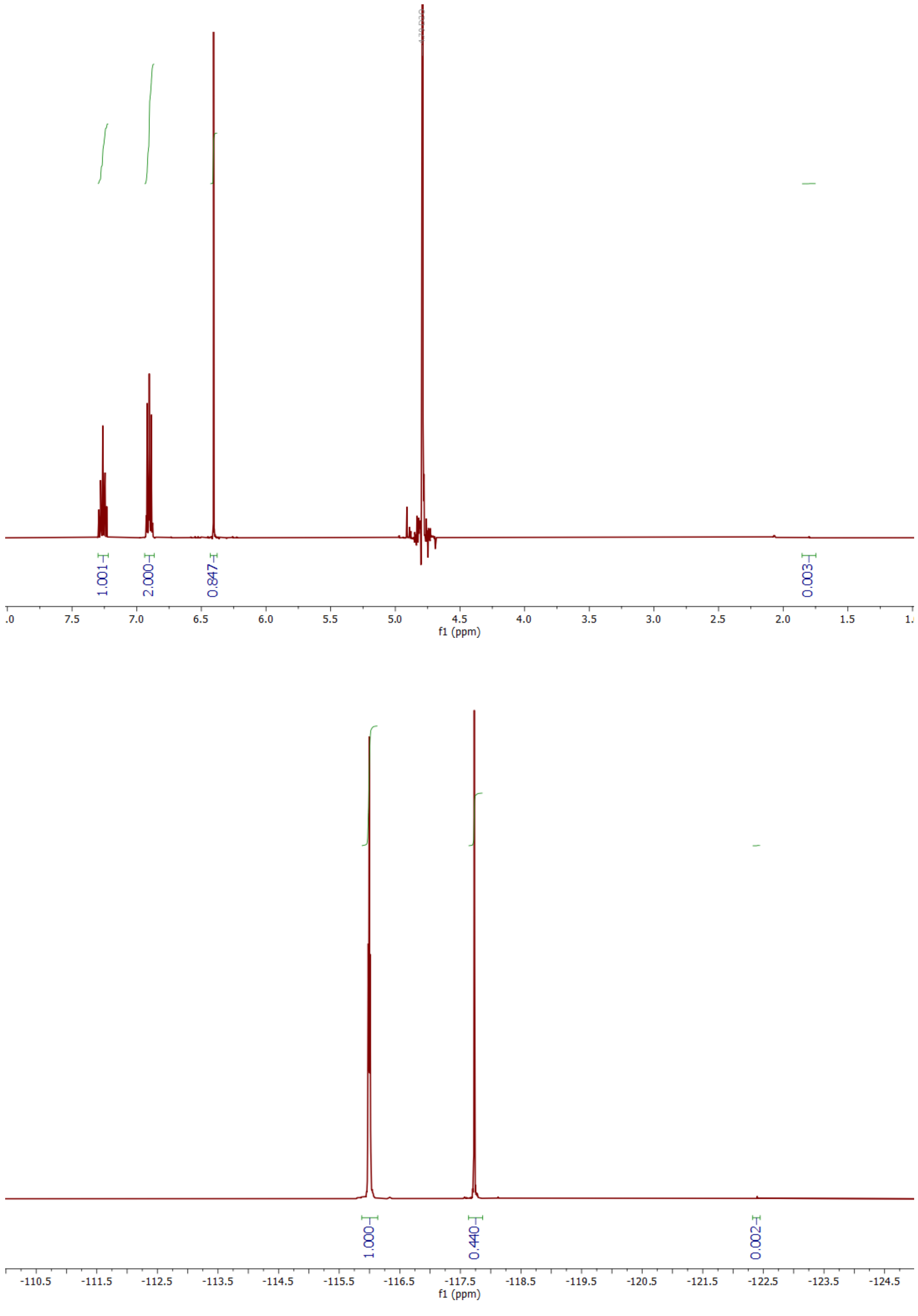

Figure S7. ^{1}H-NMR (top) and ^{19}F-NMR (bottom) spectra of **PF-MOF2**.

15.0 mg of desolvated sample was digested. In the ^{1}H-NMR spectrum, the signal of $\mathbf{FUM^{2-}}$ falls at δ_H = 6.40 ppm and accounts for two protons. The signal of AA falls at δ_H = 1.79 ppm and accounts for three protons. Thus, to have a quantitative comparison of the two species, the integral of $\mathbf{FUM^{2-}}$ (0.847) must be divided by two, giving 0.424, whereas the integral of AA (0.002) must be divided for three, giving 0.001. The $\mathbf{FUM^{2-}}$/AA ratio is therefore 424, suggesting that practically no AA is present in the MIXMOF after PSE. The two signals of DFBA fall at δ_H = 6.90 ppm (accounts for two protons) and δ_H = 7.26 ppm (accounts for one proton). Taking the value of the DFBA signal falling at δ_H = 7.26 ppm (1.000), a $\mathbf{FUM^{2-}}$/DFBA ratio of 0.424 is obtained. In the ^{19}F-NMR spectrum, the signal of $\mathbf{TFS^{2-}}$ falls at δ_F = −117.8 ppm and accounts for four fluorine atoms. The signal of DFBA falls at δ_F = −116.0 ppm and accounts for two fluorine atoms. Thus, to have a quantitative comparison of the two species, the integral of $\mathbf{TFS^{2-}}$ (0.440) must be divided by four, giving 0.110, whereas the integral of DFBA (1.000) must be divided by two, giving 0.500. A $\mathbf{TFS^{2-}}$/DFBA ratio of 0.220 is obtained. Using the values of $\mathbf{FUM^{2-}}$/DFBA and $\mathbf{TFS^{2-}}$/DFBA ratios, a $\mathbf{FUM^{2-}}$/$\mathbf{TFS^{2-}}$ ratio of 1.93 is obtained. As already explained for **PF-MOF1**, for the sake of simplicity, we report here the result that best fits with the experimental data for **PF-MOF2** as well, i.e. the one that involves ligand exchange only at defective sites, with no incorporation in the framework. The general formula $[Zr_6O_4(\mu_3\text{-}OH)_4(\mu_1\text{-}OH)_x(H_2O)_x(\mathbf{FUM})_{4.04}(\mathbf{HTFS})_{3.92-x}]$ is proposed. The following equation can be written:

$$\frac{4.04}{3.92 - x} = 1.93$$

This leads to calculate values of 1.83 for x, leading to the following proposed formula for **PF-MOF2**: $[Zr_6O_4(\mu_3\text{-}OH)_4(\mu_1\text{-}OH)_{1.83}(H_2O)_{1.83}(\mathbf{FUM})_{4.04}(\mathbf{HTFS})_{2.09}]$. Such a formula leads to calculate expected wt.% of 28.8% for $\mathbf{FUM^{2-}}$ and of 24.7% for $\mathbf{HTFS^{-}}$. The absolute concentration

of **FUM^{2-}** and **TFS^{2-}** (note that the solution is strongly alkaline, so that **H$_2$TFS** is fully deprotonated) in solution can be derived by multiplying the **FUM^{2-}**/DFBA and **TFS^{2-}**/DFBA ratios by 0.1, obtaining 0.0424 M for **FUM^{2-}** and 0.0220 M for **TFS^{2-}**. The weight % of **FUM^{2-}** and **HTFS^{-}** in the MOF can be derived by multiplying these values by 1.0 (volume of the solution) and the molecular weight of the respective anions (114 for **FUM^{2-}**, 189 for **HTFS^{-}**) and dividing the obtained values (4.83 mg for **FUM^{2-}**, 4.16 mg for **HTFS^{-}**) by the mass of the desolvated MOF digested (15.0 mg), resulting in 32.2% for **FUM^{2-}** and 27.6% for **HTFS^{-}**. These values are slightly higher than those expected from the formula proposed above. However, other scenarios where **TFS^{2-}** substitutes for **FUM^{2-}** in the framework lead to much larger discrepancies, suggesting that the proposed formula is the most reliable. To further confirm the reliability of this formula, we refer to the TG curve displayed in Figure 3 in the main text, which shows that **PF-MOF2** loses 25 wt% upon desolvation (at 280 °C) and a further 38 wt% upon degradation of the organic part of the framework (at 700 °C), when only ZrO_2 is left. Thus, a ratio of 0.49 can be calculated between the mass% of leftover ZrO_2 (37%) and that of the desolvated MOF (75%). Given that the formula [$Zr_6O_4(\mu_3\text{-}OH)_4(\mu_1\text{-}OH)_{1.83}(H_2O)_{1.83}$(**FUM**)$_{4.04}$(**HTFS**)$_{2.09}$] has a FW of 1598 g/mol, and that 6 moles of ZrO_2 per mole of MOF are expected to remain after full decomposition, a ratio of 0.46 is calculated between the expected mass of leftover ZrO_2 and that of the desolvated MOF with the above formula, confirming that the proposed formula is in line with the experimental outcomes from the thermogravimetric analysis.

ICP-OES analysis of the reaction supernatant after PSE on MOF-801

To gain deeper insight on the PSE mechanism and to quantify any zirconium leaching from the MOF during the linkers exchange, an ICP-OES analysis was performed on the reaction supernatant. The latter was collected and further worked up by adding 2% wt. of nitric acid and by bringing the total volume to 15 mL. According to the data shown in Table S2, there is no appreciable metal leaching during PSE, since the metal percentage in solution in both samples is less than 0.1%.

	**Zr (ppm)**	**V (mL)**	**%Zr_{dis}/Zr_{tot}**
**PF-MOF1**	0.598	15	0.011
**PF-MOF2**	3.667	15	0.067

Table S2. ICP-OES results from the analysis carried out on the reaction supernatant in the synthesis of **PF-MOF1** and **PF-MOF2**.

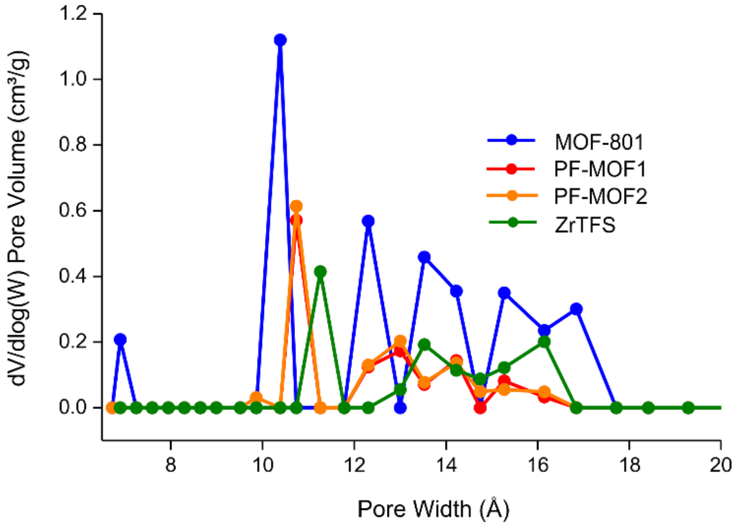

Figure S8. Pore size distribution of the four MOFs discussed in this work as derived from their CO_2 adsorption isotherms measured at T = 0 °C (2D-NLDFT method for slit pores for carbonaceous materials with heterogeneous surfaces).

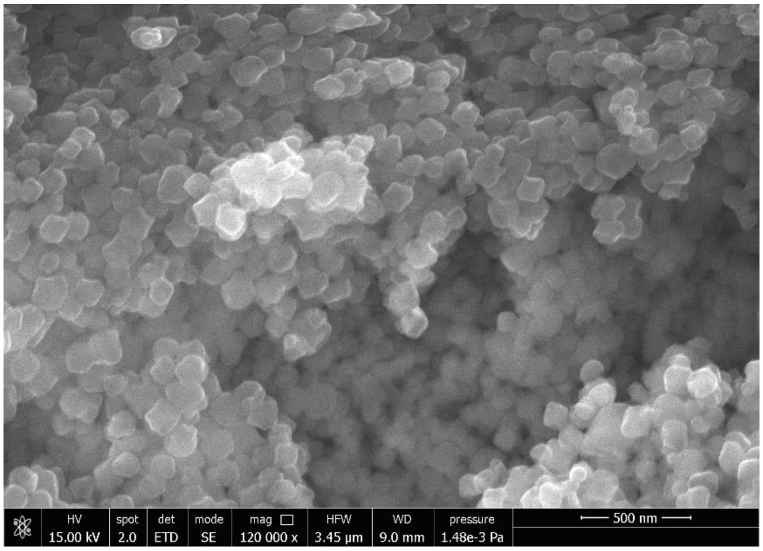

Figure S9. SEM image of **MOF-801**.

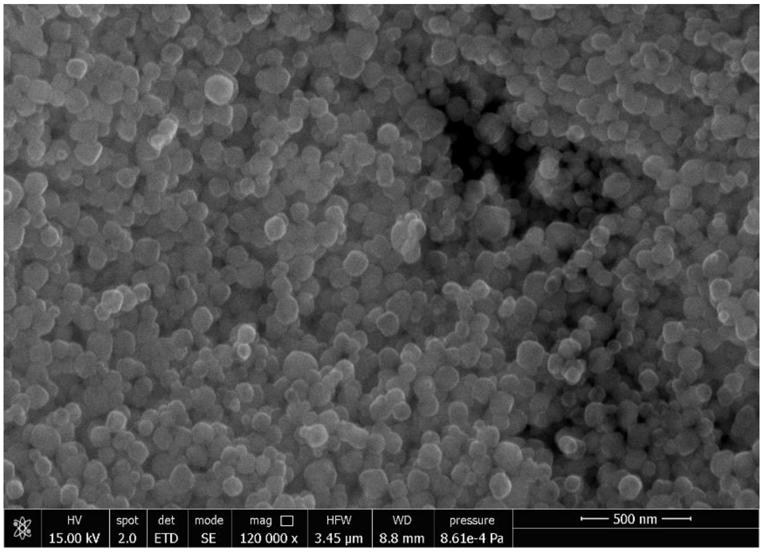

Figure S10. SEM image of **PF-MOF1**

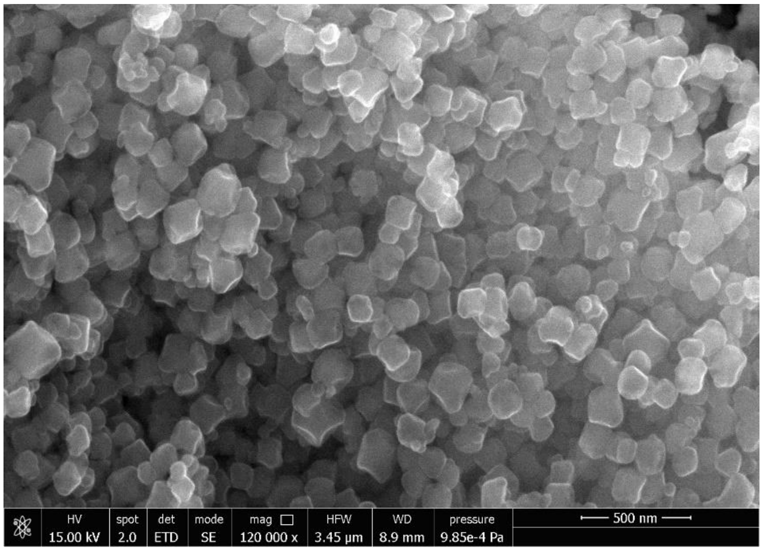

Figure S11. SEM image of **PF-MOF2**

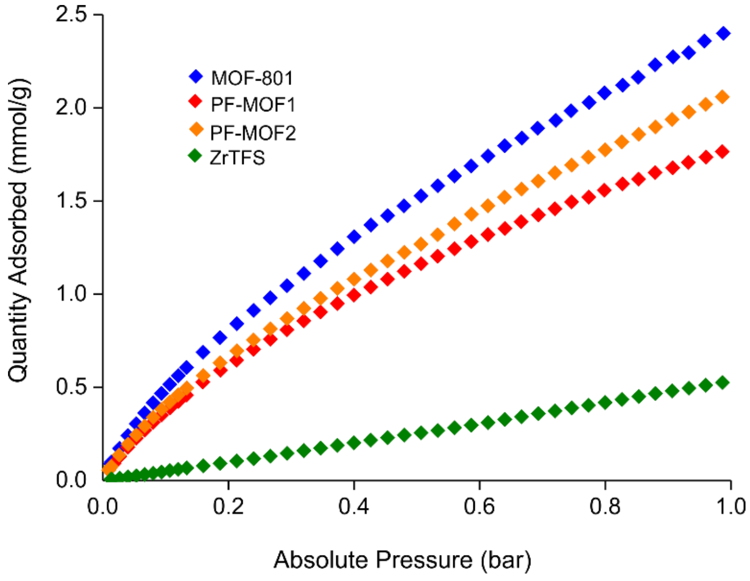

Figure S12. CO_2 adsorption isotherms measured at 25 °C on the four MOFs, with CO_2 adsorbed amounts expressed in [mmol/g] units.

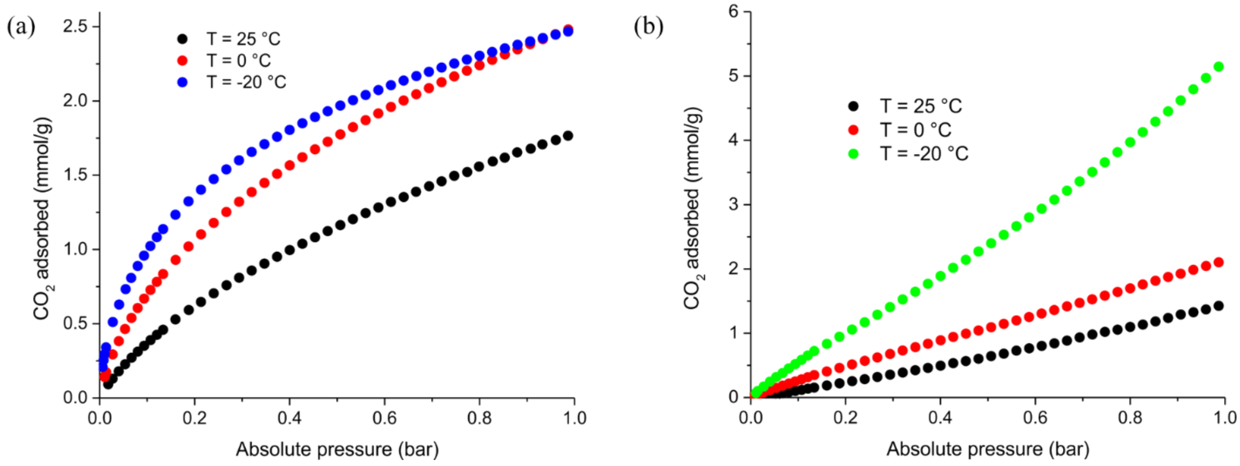

Figure S13. CO_2 adsorption isotherms of **PF-MOF1** (a) and **ZrTFS** (b) at T = −20, 0 and 25 °C.

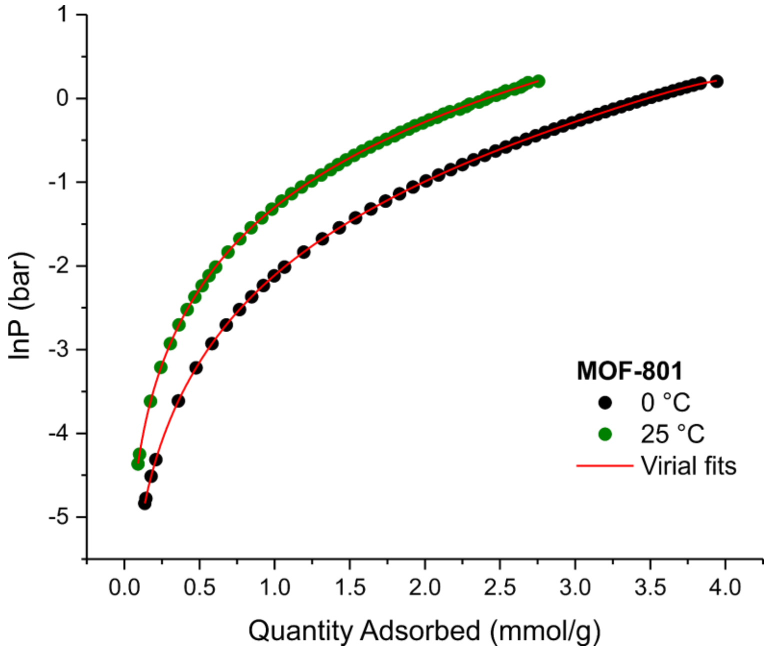

Figure S14. Virial fitting (red lines) of the CO_2 adsorption isotherms collected at 0 °C (black circles) and 25 °C (green circles) for **MOF-801.**

**Parameter**	**Value**	**Standard Error**
a_0	-3098.62022	6.92273
a_1	596.4599	13.76678
a_2	-172.15887	36.97885
a_3	17.56675	45.87883
a_4	31.08048	29.65816
a_5	-17.4914	10.33677
a_6	3.79362	1.83915
a_7	-0.30442	0.13091
b_0	8.33107	0.02393
b_1	-0.77304	0.01378
R^2	0.99999	

Table S3. Virial fitting parameters for **MOF-801.**

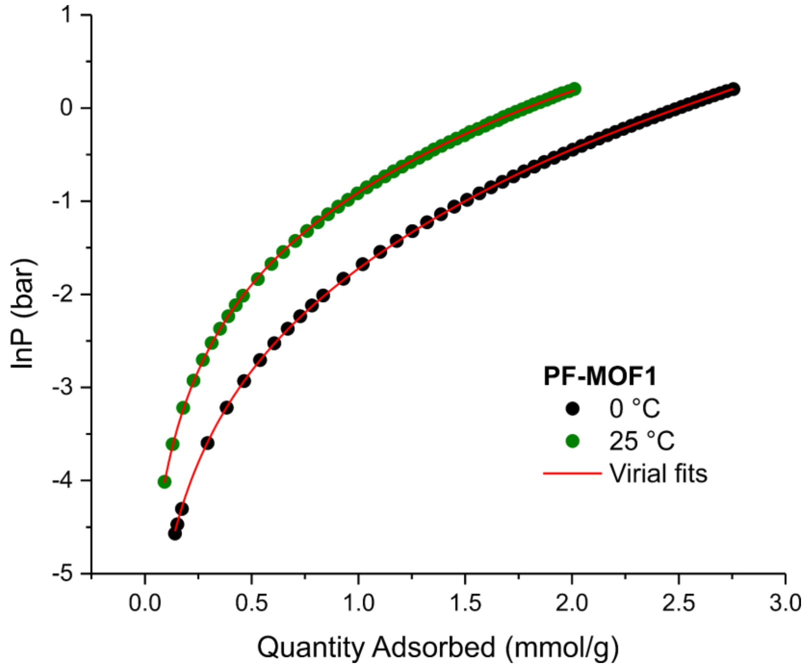

Figure S15. Virial fitting (red lines) of the CO_2 adsorption isotherms collected at 0 °C (black circles) and 25 °C (green circles) for **PF-MOF1.**

**Parameter**	**Value**	**Standard Error**
a_0	-3440.35788	14.01911
a_1	1052.15736	50.50751
a_2	-537.54222	180.85076
a_3	394.52711	304.63038
a_4	-165.68149	271.45107
a_5	35.87607	131.55613
a_6	-3.27316	32.73176
a_7	0.05118	3.27093
b_0	9.74876	0.04701
b_1	-1.71421	0.0358
R^2	0.99995	

Table S4. Virial fitting parameters for **PF-MOF1.**

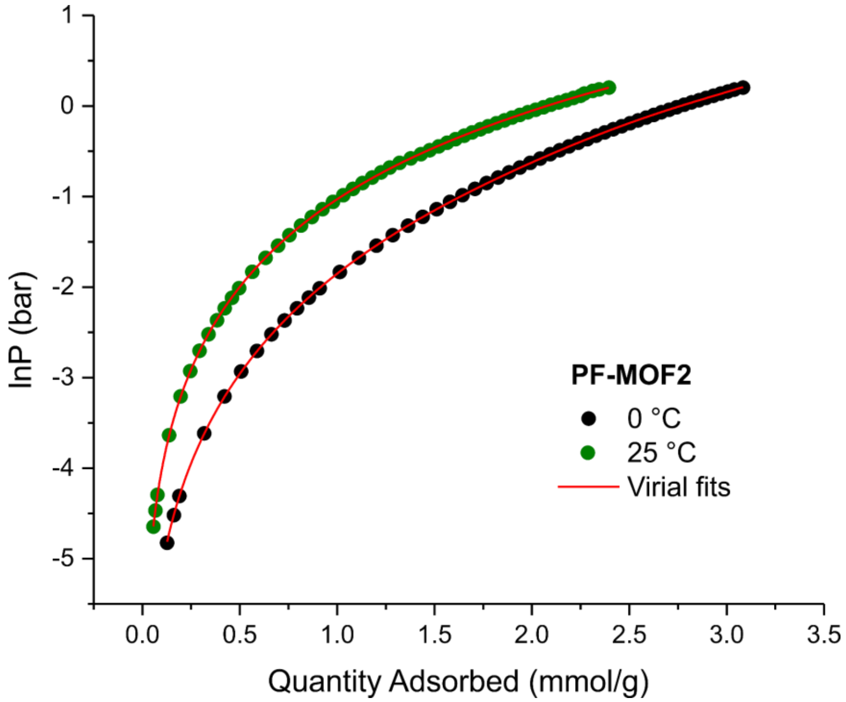

Figure S16. Virial fitting (red lines) of the CO_2 adsorption isotherms collected at 0 °C (black circles) and 25 °C (green circles) for **PF-MOF2**.

**Parameter**	**Value**	**Standard Error**
a_0	-3657.19376	7.80209
a_1	1241.87187	18.12231
a_2	-527.87117	60.19769
a_3	476.32321	96.49476
a_4	-307.92544	80.20506
a_5	123.86506	35.82496
a_6	-26.9839	8.15385
a_7	2.40171	0.74178
b_0	10.41059	0.02584
b_1	-2.46239	0.01689
R^2	0.99999	

Table S5. Virial fitting parameters for **PF-MOF2**

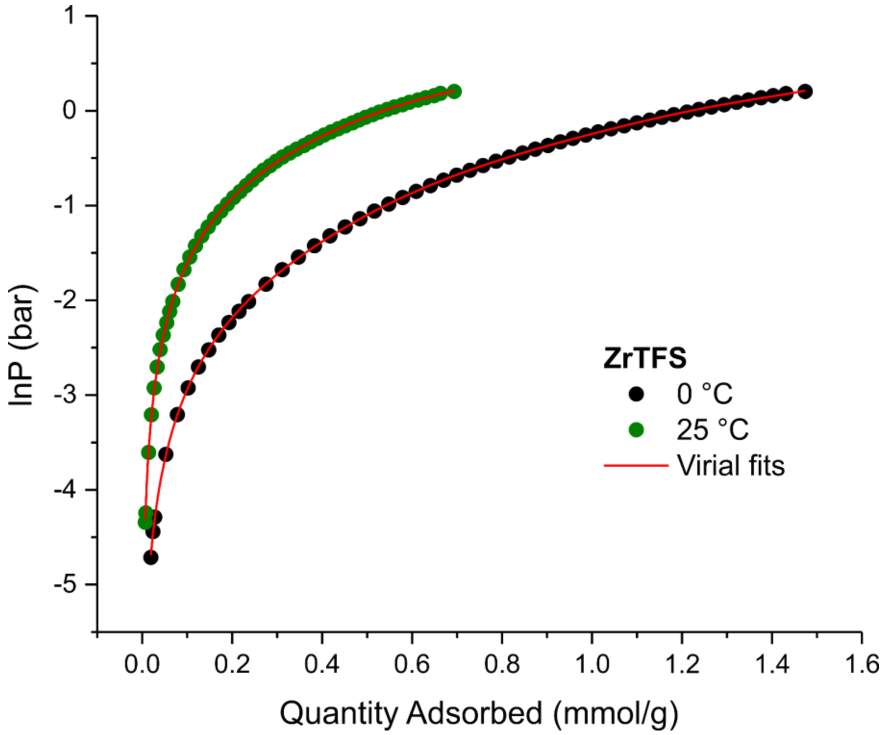

Figure S17. Virial fitting (red lines) of the CO_2 adsorption isotherms collected at 0 °C (black circles) and 25 °C (green circles) for **Zr-TFS**.

Parameter	Value	Standard Error
a_0	-3098.62022	6.92273
a_1	596.4599	13.76678
a_2	-172.15887	36.97885
a_3	17.56675	45.87883
a_4	31.08048	29.65816
a_5	-17.4914	10.33677
a_6	3.79362	1.83915
a_7	-0.30442	0.13091
b_0	8.33107	0.02393
b_1	-0.77304	0.01378
R^2	0.99999	

Table S6. Virial fitting parameters for **Zr-TFS.**

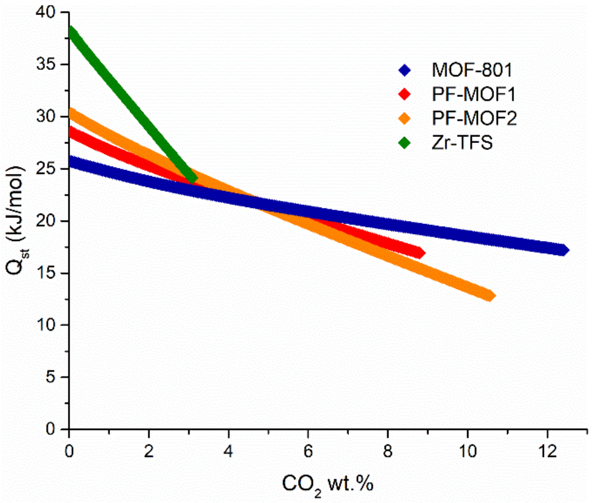

Figure S18. Plot of the isosteric heat of adsorption (Q_{st}) calculated using the parameters obtained from virial fitting of the adsorption isotherms *vs*. the CO_2 loading for **MOF-801** (blue), **PF-MOF1** (red), **PF-MOF2** (orange) and **Zr-TFS** (green).

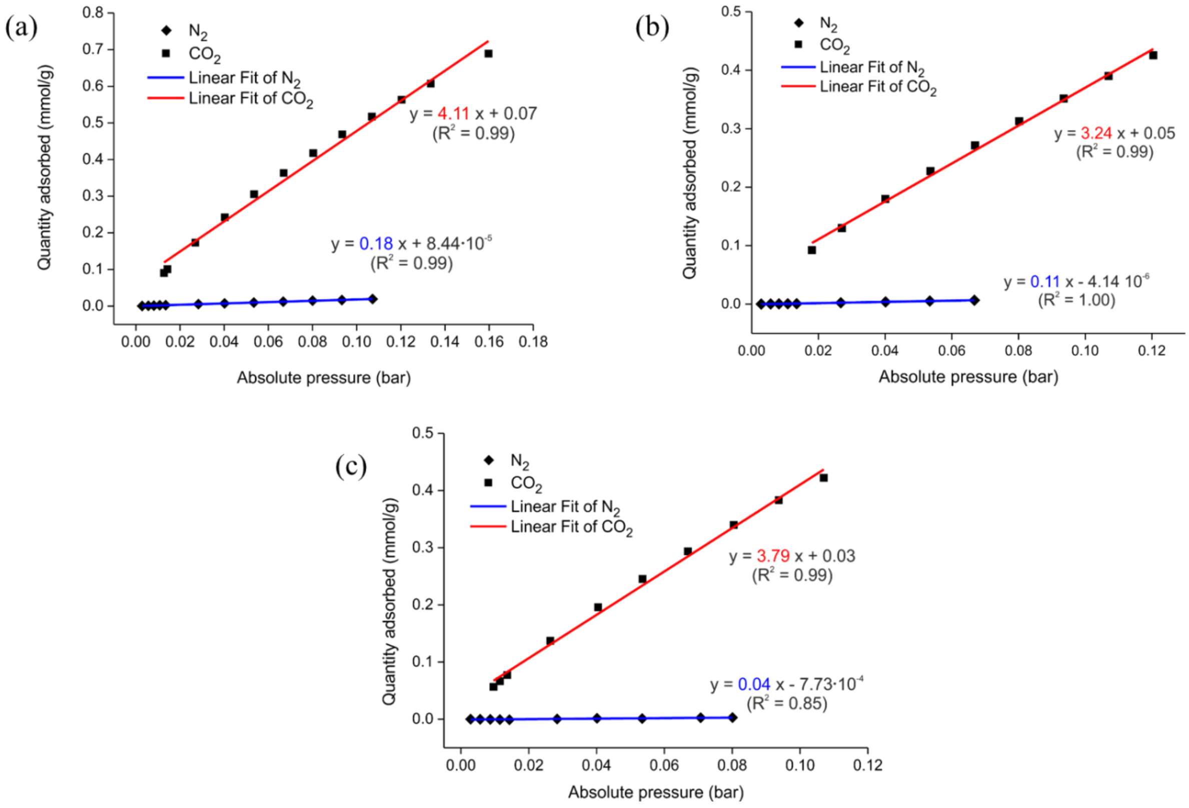

Figure S19. Isotherms linear fitting for the calculation of the CO_2/N_2 Henry selectivity at 25 °C for **MOF-801** (a), **PF-MOF1** (b) and **PF-MOF2** (c).

MOF	Q_{st} [kJ mol^{-1}]	CO_2/N_2 (15:85) selectivity	CO_2 quantity adsorbed (p = 1 bar) [mmol g^{-1}] T = 25 °C	T = 0 °C	Reference
**PF-MOF1**	29.4	30 (Henry)<br>34 (IAST)	1.6	2.2	This work
**PF-MOF2**	29.8	95 (Henry)<br>41 (IAST)	2.1	2.8	This work
**F4_UiO-66(Zr)**	22	6 (IAST)	-	1.9	[4]
**F4-UiO-66(Ce)**	25	20 (IAST)	1.5	2.5	[5]
**F4-MIL-140A(Ce)**	40	1962 (IAST)	-	2.4	[6]
**NbOFFIVE-1-Ni**	54	6528 (IAST)	2.2	-	[7]
**SIFSIX-18-Ni**	52	-	2.2	-	[8]
**SIFSIX-3-Cu**	54	> 10000 (from breakthrough analysis)	2.3	-	[9]
**SIFSIX-3-Zn**	45	1818 (IAST)	2.5	-	[10]

Table S7. CO_2 adsorption data for some representative fluorinated MOFs from the literature at comparison with our samples.

Evaluation of the CO_2 heat of adsorption on PF-MOF1 and ZrTFS using three temperature points. In order to validate the calculation of Q_{st} made using the simplified version of the Clausius-Clapeyron equation (Equation 1 in the Experimental Section), an alternative estimation of the heat of adsorption of CO_2 for **PF-MOF1** and **ZrTFS** (chosen as representative examples) has been made using three temperatures, collecting an additional isotherm at T = – 20 °C and using the differential form of the Clausius-Clapeyron equation:

$$\left[\frac{\partial(lnp)}{\partial(\frac{1}{T})}\right]_{\theta} = -\frac{Q_{st}}{R}$$

where R is the gas constant (8.314 J K^{-1} mol^{-1}). The collective isotherms are plotted in Figure S13, while the related Q_{st} fitting and calculations (at the lowest comparable coverage of ~ 1.0 wt.% and 0.3 wt.% for **PF-MOF1** and **ZrTFS**, respectively) are reported in Figure S20 below. Expectedly, the values calculated on three temperatures are identical to those reported in the main text calculated on two temperatures.

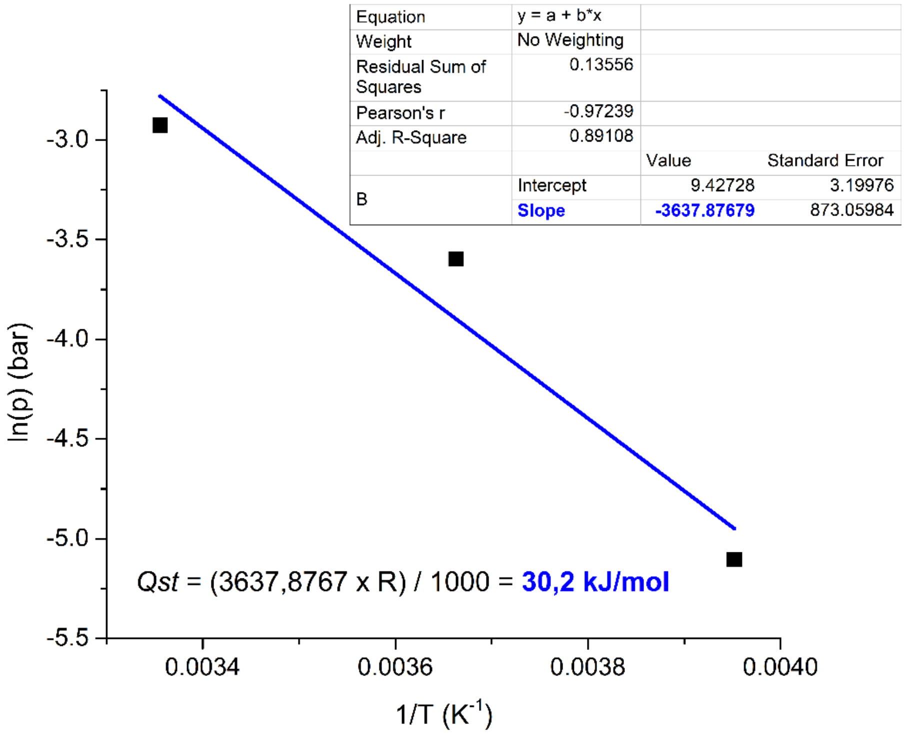

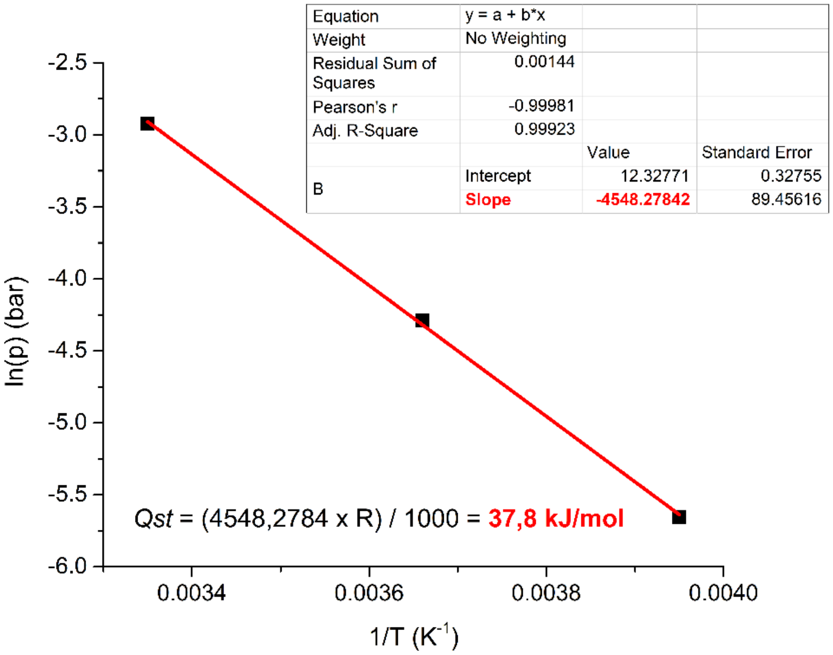

Figure S20. Linear fitting of the differential form of the Clausius-Clapeyron equation for **PF-MOF1** (top) and **ZrTFS** (bottom) at T = –20, 0 and 25 °C.

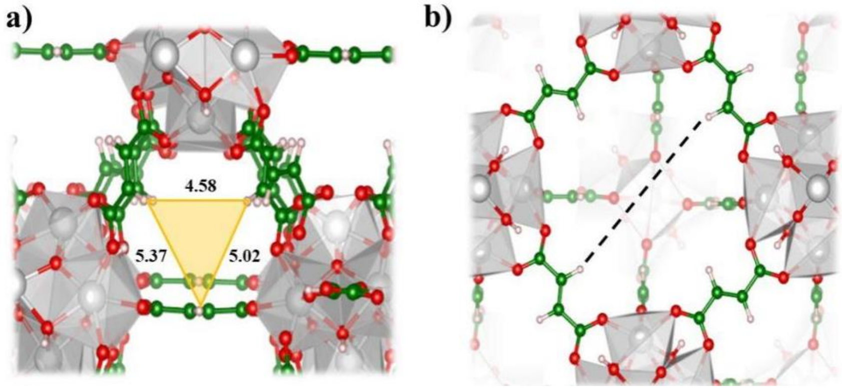

Figure S21. (a) Schematic representation of the surface of the window as the area of the triangle defined by the three hydrogen (or fluorine if **TFS**$^{2-}$ is present) atoms; (b) evaluation of the dimension of the pores simply referring to the distance (dashed line) between to opposite hydrogen or fluorine atoms. The structure of **MOF-801** is shown as representative example.

References

1 Bruker, A. X. S. *Topas V4.2*: General Profile and Structure Analysis Software for Powder Diffraction Data. Bruker AXS, Karlsruhe, Germany (**2009**).

2 Laugier, J.; Bochu, B. Gretep. Laboratoire des Matériaux et du Génie Physique de l'Ecole Nationale Supérieure de Physique de Grenoble (INPG), France (**2002**).

3 Černý, R.; Favre-Nicolin, V. FOX : A Friendly Tool to Solve Nonmolecular Structures from Powder Diffraction. *Powder Diffr.* **2005**, *20*, 359-365.

4 Zhigang Hu, Z.; Abhishek Gami, A.; Yuxiang Wang, Y.; Zhao, D. A Triphasic Modulated Hydrothermal Approach for the Synthesis of Multivariate Metal–Organic Frameworks with Hydrophobic Moieties for Highly Efficient Moisture-Resistant CO_2 Capture. *Adv. Sustainable Syst.* **2017**, *1*, 1700092.

5 D'Amato, R.; Donnadio, A.; Carta, M.; Sangregorio, C.; Tiana, D.; Vivani, R.; Taddei, M.; Costantino, F., Water-Based Synthesis and Enhanced CO_2 Capture Performance of Perfluorinated Cerium-Based Metal−Organic Frameworks with UiO66 and MIL-140 Topology. *ACS Sust. Chem. Eng.* **2019**, *7*, 394-402.

6 Shearan, S. J. I.; Jacobsen, J.; Costantino, F.; D'Amato, R.; Novikov, D.; Stock, N.; Andreoli, E.; Taddei, M., *In Situ* X-ray Diffraction Investigation of the Crystallisation of Perfluorinated Ce^{IV}-Based Metal–Organic Frameworks with UiO-66 and MIL-140 Architectures. *Chem. Eur. J.* **2021**, *27*, 6579-6592.

7 Bhatt, P. M.; Belmabkhout, Y.; Cadiau, A.; Adil, K.; Shekhah, O.; Shkurenko, A.; Barbour, L. J.; Eddaoudi, M., A Fine-Tuned Fluorinated MOF Addresses the Needs for Trace CO_2 Removal and Air Capture Using Physisorption. *J. Am. Chem. Soc.* **2016**, *138*, 9301-9307.

8 Mukherjee, S.; Sikdar, N.; O'Nolan, D.; Franz, D. M.; Gascón, V.; Kumar, A.; Kumar, N.; Scott, H. S.; Madden, D. G.; Kruger, P. E.; Space, B.; Zaworotko, M. J., Trace CO_2 Capture by an Ultramicroporous Physisorbent with Low Water Affinity. *Sci. Adv.* **2019**, *5*, eaax9171.

9 Shekhah. O.; Belmabkhout, Y.; Chen, Z.; Guillerm, V.; Cairns, A.; Adil, K.; Eddaoudi, M., Made-to-order metal-organic frameworks for trace carbon dioxide removal and air capture. *Nat. Commun.* **2014**, *5*, 4228.

10 Nugent, P., Belmabkhout, Y., Burd, S.; Cairns, A. J.; Luebke, R.; Forrest, K.; Pham, T.; Ma, S.; Space, B.; Wojtas, L.; Eddaoudi, M.; Zaworotko, M. J., Porous materials with optimal adsorption thermodynamics and kinetics for CO_2 separation. *Nature* **2013**, *495*, 80-84.
